# Coping self-efficacy mediates effects of posttraumatic distress on communal coping in parent-adolescence dyads after floods

**DOI:** 10.1017/S0954579424000567

**Published:** 2024-03-15

**Authors:** Kotaro Shoji, Charles C. Benight, Tamara Afifi, Erika D. Felix

**Affiliations:** 1 College of Nursing, University of Human Environments, Obu, Japan; 2 Department of Psychology, Lyda Hill Institute for Human Resilience, University of Colorado, Colorado Springs, CO, USA; 3 Department of Communication, University of California, Santa Barbara, CA, USA; 4 Department of Counseling, Clinical, and School Psychology, University of California, Santa Barbara, CA, USA

**Keywords:** coping self-efficacy, communal coping, posttraumatic stress symptoms, Bayesian dyadic multilevel modeling, parent-youth dyads

## Abstract

Social cognitive theory provides a framework of human agency during environmental challenges, with coping self-efficacy (CSE) as an important construct underlying adaptation. We examined two alternative models involving CSE as a mediator of the association between posttraumatic stress symptoms (PTSS) and communal coping among parent-youth dyads after severe floods using Bayesian dyadic multilevel modeling. The first model included PTSS as the independent variable and communal coping as the dependent variable (disaster distress model). The independent and dependent variables were replaced for each other in the second model (communal coping model). We used data from 485 parent-youth dyads who experienced floods between 2015 and 2016 in Texas, USA. Parents of children (69% women) aged 10–19 years old, and their oldest child (53% male; Mean age = 13.75) in that age range were recruited. We assessed PTSS, CSE, and communal coping for parents and youths. Results favored the disaster distress model over the communal coping model. In the disaster distress model, results demonstrated that CSE declines as PTSS increases, predicting decreased communal coping. This mediation effect of CSE is stronger for youths compared to parents, indicating that children’s CSE is affected more by PTSS.

## Introduction

Natural disasters, like floods, can result in devastating consequences for the livelihoods of people who live in the affected areas. People can lose their homes, household belongings, and loved ones. Furthermore, floods can result in an increased risk for posttraumatic stress disorder (PTSD), due to possible exposure to life threatening events and injury to self or loved ones. PTSD symptoms include episodes of repeated reliving of trauma, having intrusive memories, a sense of numbness, detachment from other people, autonomic hyperarousal, hypervigilance, and sleep problems (World Health Organization, 2021). Meta-analytic studies showed that the prevalence of PTSD ranged between 19.2% and 30.0% among 58,396 children and adolescents who had survived floods or earthquakes and 15.7% among survivors of floods (Chen & Liu, 2015; Rezayat et al., 2020). These studies indicate that a substantial portion of both adults and youths have a risk of developing PTSD after floods. Given this risk, it is imperative to understand how protective factors, like coping self-efficacy (CSE) and communal coping, work in families to help mitigate the risk for posttraumatic stress symptoms (PTSS).

### Social cognitive theory and post-disaster familial functioning

Social cognitive theory provides a useful framework of human agency during environmental challenges (Bandura, 1997). Benight and Bandura (2004) argued that social cognitive theory was particularly helpful for understanding how individuals manage extreme events such as natural disasters, interpersonal violence, and terrorist attacks. Self-regulation is the process where human beings utilize internal and external information (i.e., feedback) to adapt to an ever-changing environment in order to achieve desired states (Bandura, 1997). Human beings’ ability to be self-aware allows for self-evaluative judgments (e.g., “I am gaining ground on dealing with the insurance company!!!”), which are central to the self-regulation process. Coping self-efficacy is derived from self-evaluative judgments and a prime determinant of behavior (Bandura, 1997). A series of disaster studies showed the predictive power of coping self-efficacy in explaining the impact of the disaster on psychological distress (Benight & Bandura, 2004). Furthermore, Hobfoll and colleagues argued that promoting a sense of CSE is one of five essential elements for the recovery from stressful events (Hobfoll et al., 2009).

CSE refers to a belief in the capability to cope with uncertain and challenging situations (Benight & Bandura, 2004). Disasters push people’s capacity to cope in numerous ways, including dealing with the thoughts of the event itself, untangling the complexities of rebuilding, managing significant daily life disruptions, and coming to grips with the loss of loved ones. These challenging demands require effective self-regulation to meet this dynamic recovery process (Benight & Bandura, 2004). Meta-analytic findings following collective trauma demonstrated that CSE has an inverse relationship with posttraumatic distress with medium to large effect sizes in longitudinal studies (Luszczynska et al., 2009). Moreover, following major disasters including hurricanes, floods, and wildfires, research has shown CSE serves as an important mediator between disaster stress and trauma-related distress (Benight & Bandura, 2004). For example, disaster-related losses are related to lower CSE, which is further related to subsequent elevated trauma-related distress (Benight et al., 1999). In addition to these functions of CSE, it can be a factor affecting the shift from low to high levels of PTSS in a disaster context (Benight et al., 2020).

Whereas this previous research underscores the importance of individual perceptions of coping capability, it is also important to recognize that disaster survivors are often faced with coping together or communally to manage the natural disaster itself and the recovery from it. Indeed, Bandura et al. (2011) suggested that family members do not cope in isolation. Following a major disaster, the family must seek to recover by working together in a coordinated effort. Communal coping is a key component of this regulatory capacity.

### Disasters and communal coping

Floods can affect a large geographic area and population at the same time. As such, it is important to examine how people cope with the aftermath of floods as groups and parent-child dyads. Communal coping occurs when multiple people appraise a stressor as jointly “owned” and take responsibility for addressing it together (Afifi et al., 2020; Lyons et al., 1998). Communal coping can be communicated verbally (e.g., “we will get through this together”), nonverbally (e.g., displays of affection that communicate solidarity), and through actions (e.g., helping a neighbor rebuild their house after a natural disaster). Given that most people are likely to experience and cope with natural disasters in social relationships like families (Felix et al., 2020), it is important to understand how families’ communal coping efforts and PTSD are related. In addition, most research on communal coping examines couples, siblings, or whole families coping with an illness or significant stressor (e.g., Helgeson et al., 2017) or how larger communities cope communally with natural disasters and other external threats (e.g., Richardson & Maninger, 2016). Only a few studies have examined communal coping in parent-child relationships (for exceptions, see Afifi et al., 2019; Kam et al., 2017). However, to our knowledge, no studies have examined communal coping in parent-child dyads in disaster research. The present study fills these gaps in the literature.

Communal coping in parent-child relationships is particularly important because our most essential attachments and modeling of coping skills occur within the family. In addition to parents being concerned about their children’s well-being, children look to their parents for guidance, and it is essential that children know they are not alone in combatting a stressor that affects the entire family. The parent-child relationship is also unique with regard to communal coping because of the power dynamics inherent in that relationship (Afifi et al., 2006; Borelli et al., 2016). For instance, children might cope communally with a parent out of perceived obligation to them and/or the larger family collective (Kam et al., 2017). When it is used effectively, however, communal coping has been shown to be an important source of efficacy, improved mental health, and emotional validation for adolescent children (e.g., Afifi et al., 2019; Amaro, 2020).

Communal coping can improve CSE following community-wide traumas like natural disasters because it helps people feel like they are not alone in their ability to cope with adversity (Buzzanell & Houston, 2018; Musoke et al., 2022). Creating positive affect and a shared narrative of collective resolve within a community or family through communal coping can build the efficacy of the entire group and the individuals within it (Buzzanell & Houston, 2018; Richardson & Maninger, 2016). The extended theoretical model of communal coping contends that CSE is a mediator of the association between communal coping and resilience/thriving, including mental, physical, and relational health (Afifi et al., 2020). For example, Berg et al. (2008) found that married couples who engaged in greater amounts of communal coping experienced increases in the husband’s CSE regarding his prostate cancer, which, in turn, positively affected the mood of both partners.

Alternatively, it is possible that communal coping is also an outcome. Research on resilience shows that communal coping is often fostered through family members experiencing stressful circumstances and coping with them together (Afifi et al., 2016; Patterson, 2002). Thus, communal coping could be a function of post-disaster distress or vice versa. We examined these possibilities by testing models involving these two scenarios with CSE as a mediator in parent-youth pairs after floods.

### Parent-youth dyadic relationship

The associations among communal coping, CSE, and mental health have been tested primarily in the context of chronic illnesses (Berg et al., 2008). However, the same pattern should hold true theoretically regarding parents’ and adolescents’ ability to cope with a natural disaster. Children often experience natural disasters together with their parents or guardians. Children’s coping experiences with natural disasters need to be understood in conjunction with their parents’ coping experiences. In their comprehensive review, Bonanno et al. (2010) found that greater parental distress post-disaster was related to higher levels of symptoms in children. They noted that this was due in part to shared trauma exposure, but also because of the influence on family dynamics. Indeed, examination of the post-disaster family environment suggests that relationship quality (Felix et al., 2013) and communication strategies (Felix et al., 2020) can affect youth post-disaster mental health. Although prior research suggests that children are affected by a traumatic event itself and parental mental health, they did not assess how one family member’s coping may relate to the other family member’s coping. We examine cross-partner correlations (e.g., do youths who have a strong relationship between CSE and PTSS have parents who also have a strong relationship between CSE and PTSS?) with Bayesian dyadic multilevel modeling. This analytical approach allows us to examine the reciprocal relationship between parents and their adolescent children in the post-disaster recovery.

### Present study

Extreme weather events are becoming more frequent and severe, with floods and storms being the most common over the past decade (Centre for Research on the Epidemiology of Disasters, 2022). During a one-year period, the U.S. Federal Emergency Management Association (FEMA) made six major disaster declarations for Texas with 159 out of 254 counties receiving FEMA declarations for individual and/or public assistance.

**Figure 1.**
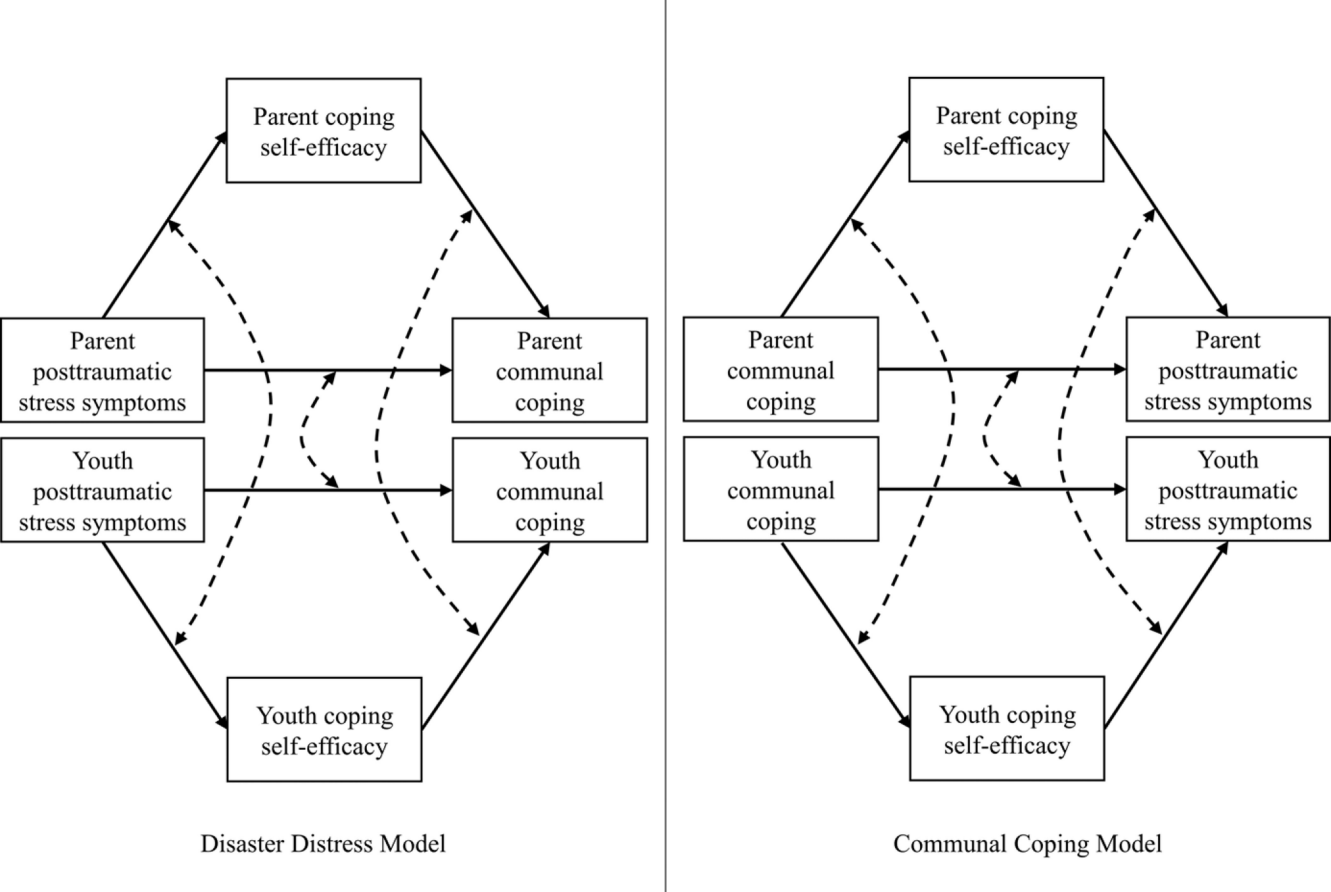
Disaster distress model and communal coping model. *Note*. The dotted lines indicate intervariable cross-pair correlations.

The present study surveyed parents and youths in Texas following a year of devastating floods and examined two models exploring the associations between PTSS, CSE, and communal coping with parent-youth dyads. Specifically, the two alternative models included CSE as a mediator between either PTSS and communal coping or communal coping and PTSS (Figure 1). Bandura (1997) argued that human beings develop CSE beliefs based on critical feedback (e.g., posttraumatic distress) suggestive of effective or ineffective coping. Enhanced efficacy beliefs empower the person to continue to engage in active coping efforts and persevere when obstacles are encountered (Bandura, 1997). Given the demands for a family to work together following a major disaster, the mediation process of PTSS through CSE would enhance communal coping efforts when efficacy is elevated and diminish communal coping when CSE declines. This disaster distress mediation model was tested with PTSS as an independent predictor, CSE as a mediator, and communal coping as the dependent variable (Figure 1).

Alternatively, it is conceivable that the social support gained through communal coping can drive self-efficacy beliefs (Schwarzer & Knoll, 2007). Social support can enhance self-efficacy through two primary mechanisms. First, CSE increased through verbal persuasion of one’s trusted social network (“You can do it, I know you can!”). Second, human beings are social beings where we learn through watching others. Communal coping may enhance one’s CSE through this modeling process as one family member observes another successfully managing post-disaster challenges (Bandura, 1997; Warner et al., 2015). Thus, we examined communal coping as the independent predictor, CSE as a mediator, and PTSS as the dependent variable in the communal coping model.

We examined and compared these models with Bayesian dyadic multilevel modeling. The models included parent and youth components (Figure 1). To evaluate the similarities and differences between parents and youths, we examined cross-pair correlations between parents and youths. Cross-pair correlations provide evidence for whether parents and youths show the same strengths of the intervariable relationships. Specifically, cross-pair correlations offer evidence for whether the associations between PTSS and CSE, between CSE and communal coping, and between PTSS and communal coping would be consistent between parents and youths. Inconsistent cross-pair correlations could indicate different dynamic patterns; for example, youths’ CSE perception is more affected by PTSS compared to parents. We hypothesized that the coefficients of the mediation effect of CSE in the relationship between communal coping and PTSD symptoms would be greater than zero for both parents and youths. If it is greater than zero, the mediation effect would be supported. For the model comparison, we had two alternative hypotheses: (1) Disaster distress model would be a better model than communal coping model, and (2) communal coping model would be a better model than disaster distress model. We evaluated the model comparison using a Bayes factor. A Bayes factor greater than three would indicate that disaster distress model is superior. Conversely, a Bayes factor smaller than one would indicate in favor of communal coping model. Furthermore, we expected that there would be meaningful cross-pair correlations although we did not have a specific hypothesis about which associations are correlated between parents and youths.

## Method

### Procedure

Following IRB approval, we began following the Memorial Day Weekend flood of 2015 that affected 44.5% (113) of counties in Texas. Another destructive flood occurred (Halloween Weekend Flood 2015) shortly after recruitment began. IRB was modified to ask about both floods (only one person participated before this modification). During recruitment, additional flooding and storms occurred, including the April 2016 flood that affected Houston and surrounding areas. This made it prohibitive to ask exposure questions repeatedly for each flood, as families could have been affected by multiple floods. Therefore, IRB was modified to ask about the “flood most stressful to you,” and participants could indicate “Memorial Day Weekend 2015,” “Halloween Weekend 2015,” “April 2016,” or “Other” and specify which flood. Recruitment included distributing flyers at local schools, community events, and shopping centers; advertising in electronic newsletters from local schools; posting flyers in the community; door-to-door recruitment in affected neighborhoods; advertising in social media forums, newspapers, and online ads; and telephone recruitment. To reach the desired dyadic sample size, we also used an opt-in panel obtained through Qualtrics, following the April 2016 flood and severe weather. Recruitment ended in March 2017. The average time since the disaster at survey completion was 406.33 days (*SD* = 162.79). All participants completed their surveys online and received a small incentive for participation.

### Participants

Parents (*n* = 581) of children aged 10–19 years old and their oldest child in that age range (*n* = 510) were recruited in Texas. We excluded 26 parents and 24 children from our final dataset who reported their most stressful flood experience was “other” due to the wide range of past floods reported (e.g., a 2010 flood, Hurricane Katrina, etc.). As the current study is dyadic, we also excluded parents for whom their child did not participate or had incomplete data. The final sample included 485 parent-child dyads. Table 1 displays the demographic information for the sample. The median household income was $60,001–$70,000. Mean child age was 13.75 years (SD = 2.56). Most parents (68.6%) described their family as continuously intact (traditional nuclear family). United States Census data for Texas adjusted for 2016 showed the population to be 42.6% White (not Hispanic), 39.1% Latinx, 12.6% African American, 4.8% Asian, 0.1% Pacific Islander, and 1.0% Native American.

### Measures

#### Communal coping

To measure how families were coping together following the flood, study participants completed four items from the Communal Coping Scale (CCS; Afifi et al., 2006) adapted to fit the context of the flood. Parents and youth reported the extent to which their family engaged in certain positive communal coping behaviors when confronting problems related to the flood. A sample item includes “We talk through our problems together and attempt to come to solutions as a family.” Responses ranged from 1 (*strongly disagree*) to 7 (*strongly agree*) and were averaged to create a total score, with higher scores indicating greater communal coping. Internal consistency estimates were α = .89 for parents and *α* = .92 for adolescents.

#### Coping self-efficacy-trauma (CSE-T)

This measure has been used following disasters and a variety of traumas to assess participants’ perceived ability to cope with different trauma-related challenges and posttraumatic symptoms (Benight et al., 2015). Across different samples of people who experienced trauma, the CSE-T showed measurement invariance, good test-retest reliability, good internal consistency (αs = .94 – .96), and strong criterion validity (Benight et al., 2015). We used 12-items from an earlier version of the measure provided by the measure authors. Participants reported how capable they think they are to successfully deal with the specific demands of disaster recovery (currently, not as it was during the flood). Sample items included “express my feelings about what happened” and “dealing with personal losses caused by the flooding.” Participants respond on a 7-point Likert scale ranging from 1 (*not at all capable*) to 7 (*totally capable*). This study had strong internal consistency, *α* = .94 for parents and *α* = .95 for youth.

#### Posttraumatic stress symptoms (PTSS)

Parents completed the Impact of Event Scale (IES-6; Thoresen et al., 2010), and youth completed the Children’s Revised Impact of Event Scale-8 (CRIES-8; Yule, 1997). Participants were asked to indicate, with respect to the flood that was most stressful to them, how much they were distressed or bothered during the past seven days by each difficulty listed. Response options were 0 (*not at all*) to 4 (*extremely*), with a total score representing the sum of responses. The IES-6 is a 6-item measure of PTSS derived from the IES-Revised (Weiss & Marmar, 1997). It contains two items each assessing intrusion (e.g., “Other things kept making me think about it”), avoidance (“I tried not to think about it”), and hyperarousal (“I had trouble concentrating”). The IES-6 correlated highly (pooled correlation = 0.95) with the IES-R in four different samples of individuals exposed to a traumatic event, across gender, age, type of trauma, and trauma severity, and has good internal consistency (*α* = .80; Thoresen et al., 2010). The CRIES-8 is used with children aged 8 years and older and measures intrusion and avoidance. Studies support the validity and reliability of the CRIES-8 (Yule, 1997). Our data yielded α = .95 for both the parent and youth scales.

### Analytical strategy

We used Bayesian dyadic multilevel modeling to examine the disaster distress model and the communal coping model using an R package brms (Bürkner, 2017). A Bayesian approach allows uncertainty in results with posterior credible intervals (CI) as opposed to a fixed point (e.g., an unstandardized coefficient) and allows for integrating the prior knowledge into a model. We modeled random slopes of independent variables for each subject and used 30,000 post-warmup iterations (iterations = 7,500, warmup = 3,750, chains = 32, thinning = 4). The initial starting value was fixed to 0 for efficiency. A 95% credible interval was calculated for each association between the variables based on the posterior distribution using the No-U-Turn Sampler (an extension of a computer simulation of probability distributions, Hamiltonian Monte Carlo; Homan & Gelman, 2014). The posterior CIs are calculated based on the prior information and the data using Markov chain Monte Carlo. The 95% credible intervals mean that the effect has a 95% probability of falling within the range. Individual parameters were interpreted based on the 95% CIs. The 95% CIs that crossed zero suggest not a meaningful parameter, indicating that no effect or no relationship is a possibility.

We compared the models using a Bayes factor. A Bayes factor greater than three indicates evidence in favor of the disaster distress model over the communal coping model (Wetzels et al., 2011). The testing of the within-model hypotheses was conducted using posterior probability. We calculated the coefficients for the mediation effect of CSE by multiplying a beta value for the relationship between PTSS and CSE by a beta value for the relationship between CSE and communal coping. We further computed the posterior probabilities of the coefficients for the mediation effect of CSE that were greater than certain values (e.g., .01, .02, .03). We reported results of the Bayesian analysis based on Kruschke’s (2021) recommendations (see Supplemental Material 1 for the R codes for this study). Additionally, we calculated cross-pair correlations to test and compare specific alternative hypotheses.

Scholars have challenged the use of a mediation analysis in a cross-sectional design. They argued that estimates are likely to be biased in a cross-sectional mediation analysis intending to test a longitudinal relationship (Maxwell & Cole, 2007; Maxwell et al., 2011). Maxwell et al. (2011) demonstrated that a cross-sectional mediation analysis is likely to be biased if the model expresses partial mediation in an autoregressive process that is stationary and in equilibrium. However, it should be noted that their demonstration might not apply to general models involving indirect and direct effects or models involving relatively shorter duration (e.g., a few hours apart on the same day) between an independent variable and a mediator and between a mediator and a dependent variable (Shrout, 2011). Shrout (2011) argued that a cross-sectional mediation model can be useful if a model is based on a well-founded theory describing causal pathways. Although our analysis is cross-sectional, we constructed our models based on social cognitive theory describing the role of self-efficacy as a potential mediator, aiming to depict a short temporal mediation process. In addition, our Bayesian approach would be highly informative about the parent-child dynamics in the recovery from traumatic experiences while controlling for within-family variability due to the scarcity of data in this field. Our Bayesian analysis is advantageous for the model comparison using a Bayesian factor, which is a ratio of probabilities for two alternative models. Further, Bayesian modeling is advantageous because it can calculate the probabilities of mediation effects.

#### Model evaluation

We tested between-model comparisons and specific within-model hypotheses. For the between-model comparison, we compared the disaster distress model and the communal coping model to determine which one better explains families’ post-disaster coping process. Additionally, we tested specific hypotheses about the mediation effect of CSE in the relationship between PTSS and communal coping.

#### Distribution of the dependent variables

**Figure 2.**
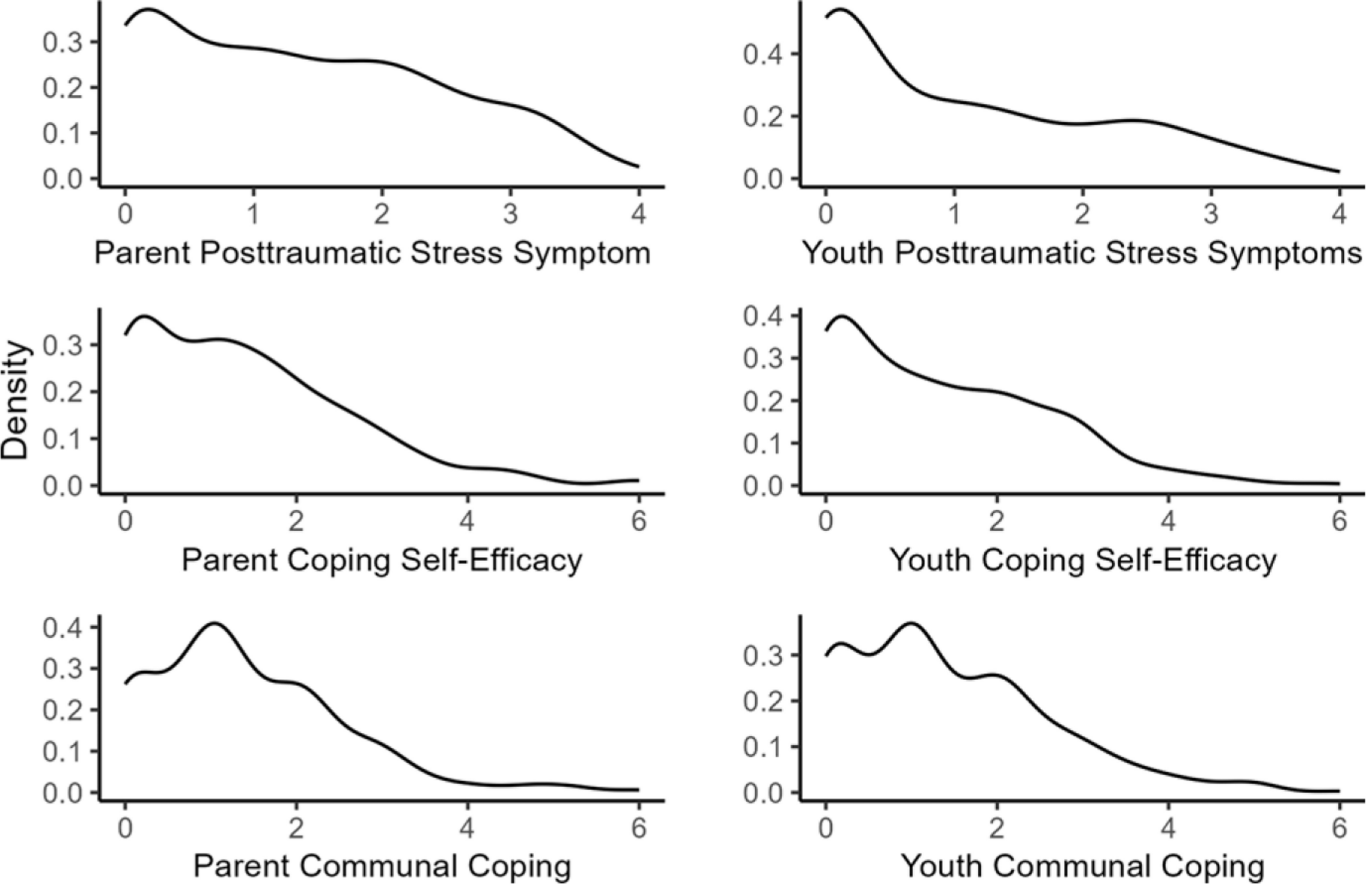
The distributions of the study variables. *Note*. Scores for CSE and communal coping were reversed.

We visually inspected a potential distribution of the study variables by density plots (Figure 2). Distributions of PTSS scores are usually positively skewed with a lot of low scores and relatively fewer high scores. The visual inspection of the distribution for PTSS confirmed this trend; thus, we decided to use the hurdle gamma distribution for both parents and youths. Distributions of CSE and communal coping are typically negatively skewed with many high scores and fewer low scores. We reverse-coded them to convert their distributions to a positively skewed distribution and used the hurdle gamma distribution for them as well. Thus, CSE was labeled as lack of CSE for the subsequent analyses.

#### Priors

A Bayesian analysis can integrate prior knowledge about the data in a model as priors. In our analysis, the priors were set up for regression coefficients of the associations between the independent variables and the dependent variables, intercepts, standard deviations, and the shape of the distribution. Table 2 displays a list of the priors used in the model. We used coefficients calculated in the previous studies as informative priors for regression coefficients when enough studies were available. When there were not enough such studies, weakly informative priors were used. Based on Gelman’s (2020) recommendations, the weakly informative prior was *student t (3, 0, 1)* for the coefficients, intercepts, and standard deviations. Finally, we performed a sensitivity test by running the model with weakly informative priors to demonstrate influences of the priors.

### Missing data

We excluded 33 dyads who did not respond to any items of the measures of CSE-T, PTSS, or communal coping. Thus, we performed the subsequent analyses based on the final 452 dyads. Of those 452, missing data comprised 2.3% of the study variables. The missing data were imputed with the random forest imputation algorithm using the R package “missForest” (Stekhoven & Bühlmann, 2012).

## Results

### Bayesian dyadic multilevel modeling

We conducted Bayesian dyadic multilevel modeling to test and compare the disaster distress and communal coping models. We first analyzed the disaster distress model.

#### Disaster distress model

**Figure 3.**
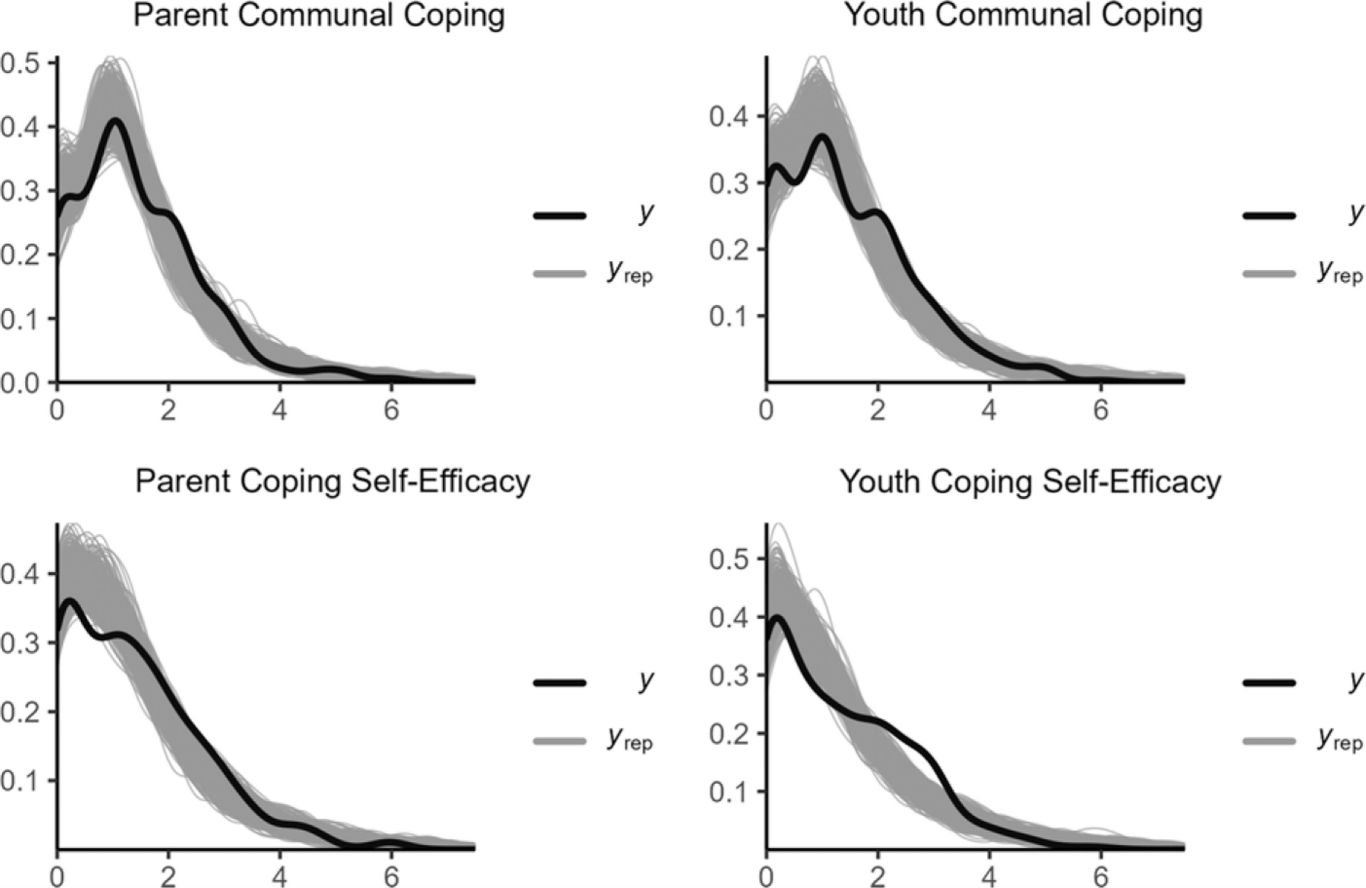
The posterior distributions of the outcomes in disaster distress model.

For the disaster distress model, the MCMC converged with $\bar{R}$ values smaller than or equal to 1.01 for all parameters (Table 3; see the visual presentations of the model convergence in Supplemental Material 2). The model had stable parameter estimates with all bulk ESS values greater than 3,200 (acceptable bulk ESS >100 times the number of chains; Clark, 2021) and tail ESS values greater than 3,000 (acceptable tail ESS > 10% of the total posterior samples). The visual inspection of the posterior distribution of the outcomes showed that the model sufficiently described the data (Figure 3). Table 3 displays the central tendency and 95% CIs for the parameters in the model. Results showed that the 95% CIs for the associations between PTSS and lack of CSE and between lack of CSE and communal coping did not include 0 for both parents and youths. However, the 95% CIs for the relationship between PTSS and communal coping crossed 0 for both parents and youths, indicating that the pathway from PTSS to communal coping was not a meaningful association. We next examined the communal coping model.

#### Communal coping model

**Figure 4.**
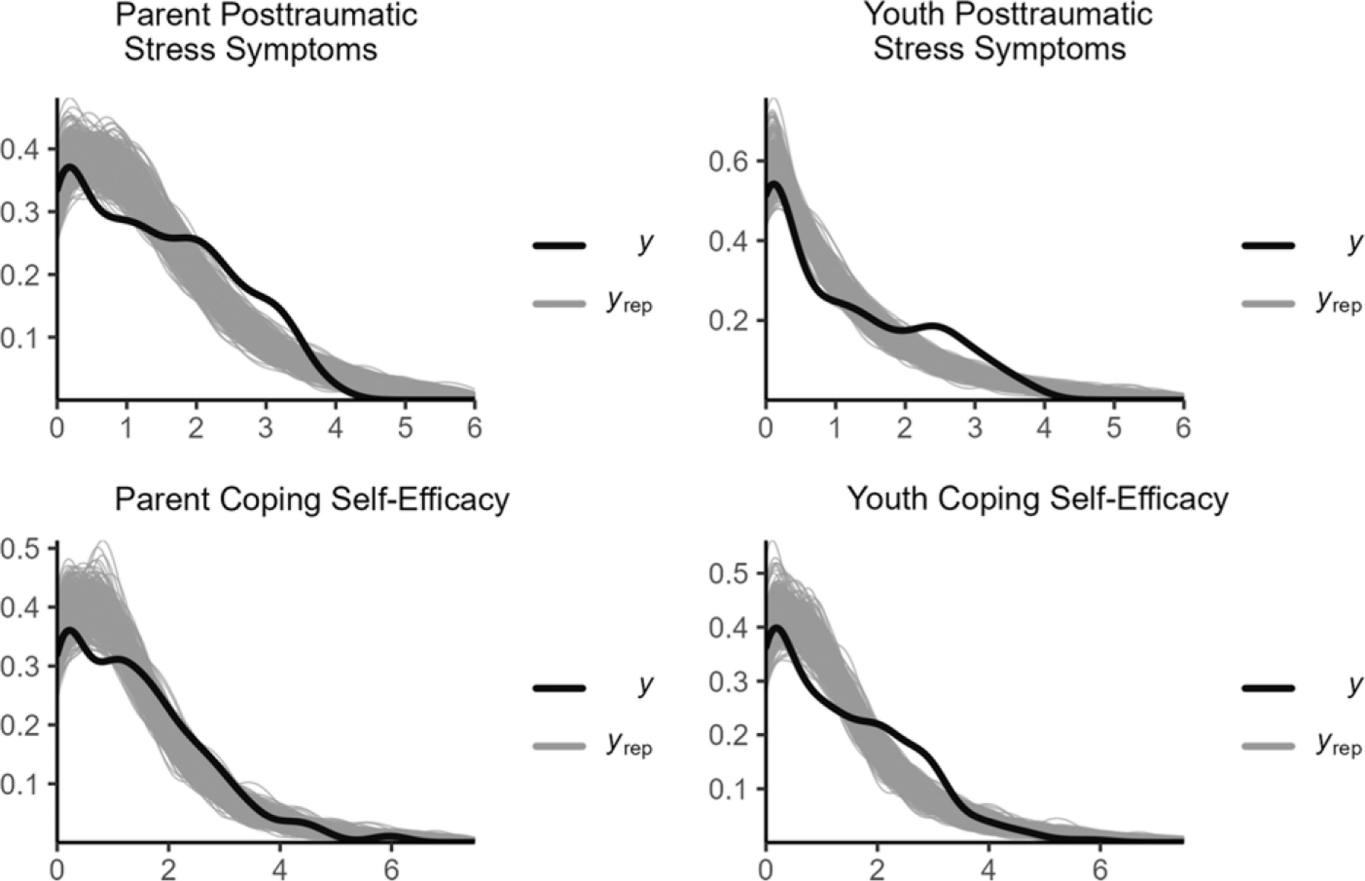
The posterior distributions of the outcomes in communal coping model.

The MCMC for the communal coping model converged well with all $\bar{R}$ values less than 1.01 (Table 4; see the visual presentations of the model convergence in Supplemental Material 3). The model showed stable parameter estimates with bulk ESS values greater than 32,00 and tail ESS values greater than 3,000 for all parameters. The posterior distributions of the outcomes sufficiently mimicked the data (Figure 4). Table 4 shows the central tendency and the 95% CIs for the parameters. The 95% CIs showed that the association between communal coping and lack of CSE and between lack of CSE and PTSS for both parents and youths did not include 0. The relationship between communal coping and PTSS crossed 0 in the 95% CIs, indicating that this pathway was not meaningful.

#### Model comparison

We compared the models using a Bayes factor. Results showed that the Bayes factor favored the disaster distress model over the communal coping model (Bayes factor = 20684120710 19927492974333237657600.00; Bayes factor >3 indicates disaster distress model superiority). Thus, we selected the disaster distress model as a better model, and the subsequent analyses focused on this model.

#### Intercorrelations of the parameters

Table 5 shows the intercorrelations of the parameters and the cross-pair correlations of the parameters for the disaster distress model. Results showed that the association between the intercepts of youth communal coping and parent communal coping did not cross 0 in the 95% CIs, indicating that youth communal coping tended to be higher when parent communal coping was higher. Similarly, the 95% CIs did not include 0 for the associations between the intercepts of youth lack of CSE and parent lack of CSE. This association indicated that youth CSE was lower when parent CSE was also lower. Furthermore, for both parents and youths, as lack of CSE was getting lower, the association between lack of CSE and PTSS became higher (more positive).

#### Mediation effect

**Figure 5.**
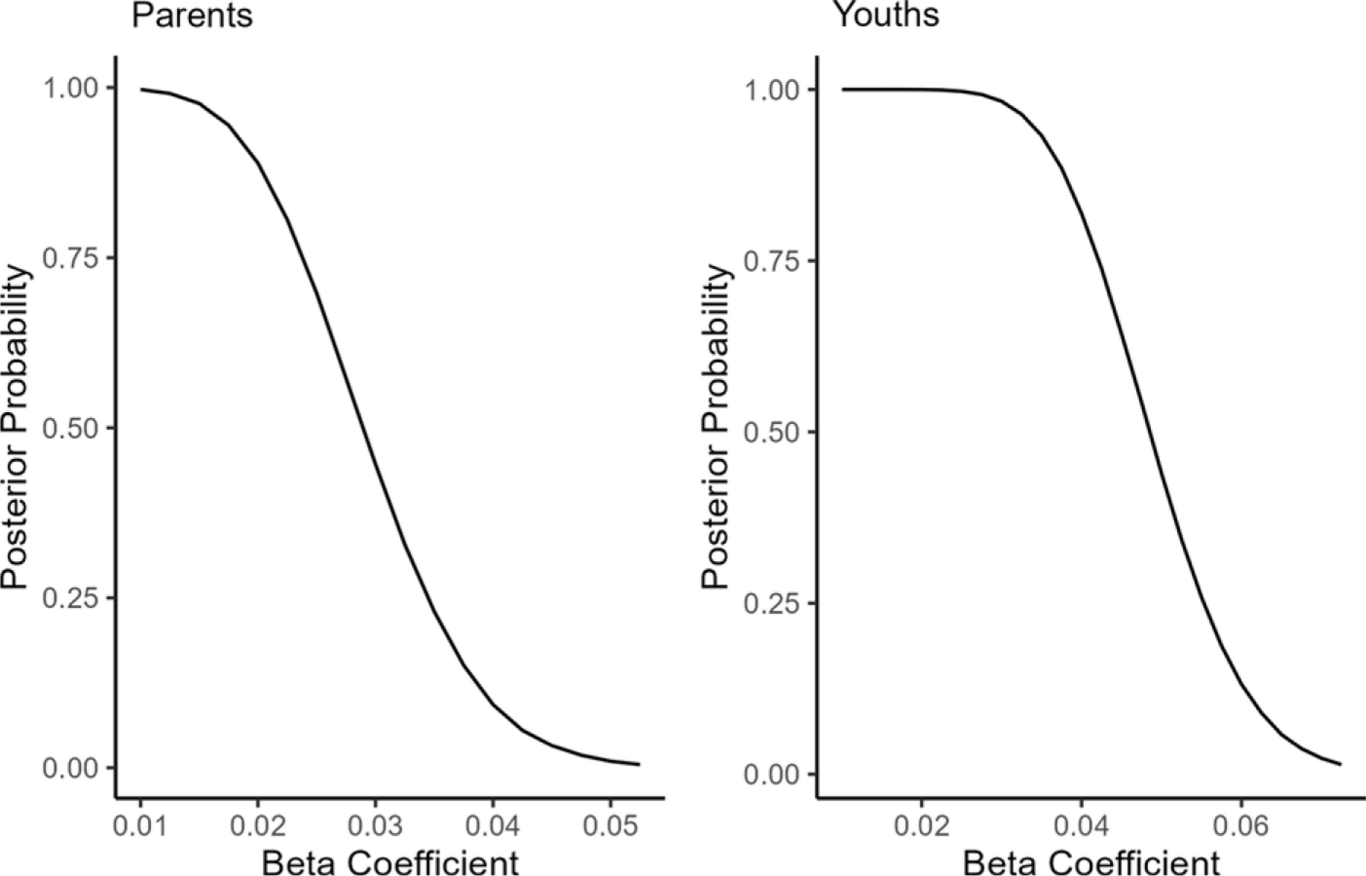
The posterior probability for the mediation effect of CSE in the relationship between PTSS and communal coping in the disaster distress model. *Note.* The values on the *y*-axis indicate the probabilities of values of the coefficient or greater.

We tested the hypothesis for the mediation effect of lack of CSE in the relationship between PTSS and communal coping for parents in the disaster distress model. Figure 5 shows the posterior probability of the coefficients (β) greater than the values on the *x*-axis. The posterior probability of the coefficients greater than .01 was .997. As the coefficient increased, the probability gradually decreased (e.g., .446 for β > .030 and .010 for β > .050). Similarly, we tested the hypothesis for the mediation effect of lack of CSE in the relationship between PTSS and communal coping for youths in the disaster distress model. Figure 5 shows the posterior probability of the beta coefficients (β) greater than the values on the *x*-axis. The probabilities of the coefficients greater than .01 was 1.00 and the coefficients greater than .03 was .993. As the coefficient increased, the probability decreased (e.g., .439 for β > .050 and .023 for β > .070). These results revealed evidence of the mediation effect of lack of CSE in the relationship between PTSS and communal coping in the form of clear probabilities.

We conducted an additional test for the difference in the magnitude of the mediation effect of lack of CSE in the relationship between communal coping and PTSS between parents and youths using a Bayes factor. Results indicated that the mediation effect of lack of CSE was stronger for youths than that for parents (95% CIs [.001, .038], Bayes factor = 25.03, posterior probability = .96).

#### Sensitivity test

**Figure 6.**
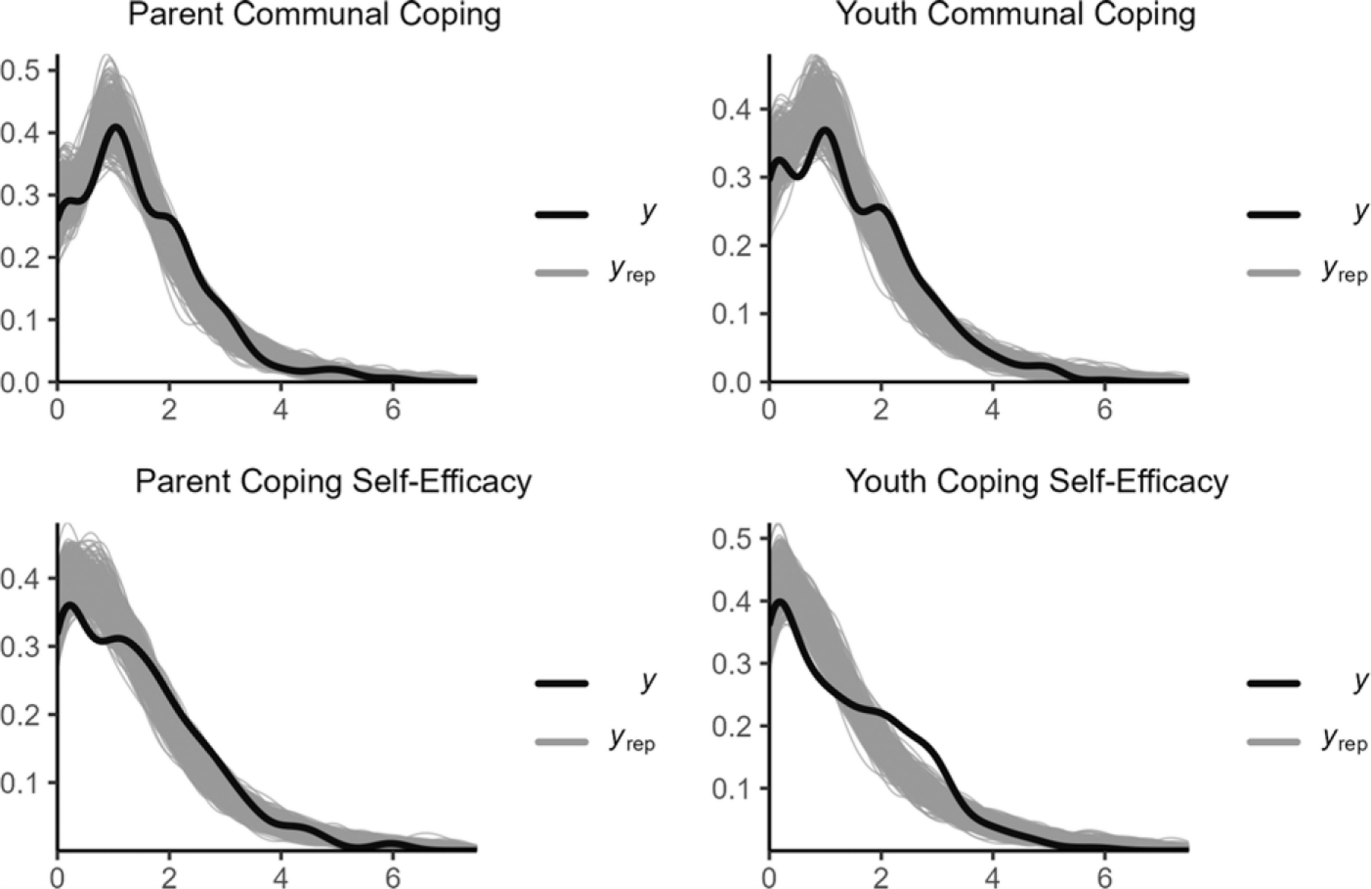
The posterior distributions of the outcomes in the sensitivity test.

To show the differences in results between using informative priors and weakly informative priors, we ran the disaster distress model with weakly informative priors with the same set up for other specifications of the model. We used the *gamma(1, 1)* prior for the shape of the distribution. Based on Gelman’s (2020) recommendations, the priors for the coefficients, intercepts, and standard deviations were *student t (3, 0, 1)*. Results showed that the MCMC converged well with R-hat values smaller than 1.01 for all parameters (Table 6; see the visual presentations of the model convergence in Supplemental Material 4). The ESS values (>10,000) indicated that the model had stable parameter estimates. The posterior distributions of the outcomes sufficiently described the data (Figure 6). Table 6 shows the central tendency and the 95% CIs for the parameters. The 95% CIs indicated the results were consistent with the results from the model with informative priors with slight differences for all parameters. A Bayes factor indicated in favor of the model with informative priors over the model with weakly informative priors (Bayes factor = 1889912830.43). These results indicated that the findings of the present study were not biased by the priors we used. Instead, the informative priors resulted in a more robust estimation.

### Descriptive statistics

We calculated correlations among the study variables with a Bayesian approach using an R package brms (Bürkner, 2017, 2018, 2021). We standardized all variables, fixed the intercepts to 0, and fixed the sigma to 1. We used a gaussian distribution for distributions of the dependent variables; we ran 4 chains with 2,000 iterations and 1,000 warm-ups (4,000 post-warmup iterations). In these analyses, the original coding was used for CSE, so that higher CSE scores indicated higher CSE levels. Table 7 displays 95% credible intervals and point estimates of the correlation between the study variables. There were some notable associations. The posterior distribution showed that there was a 95% probability of the effect between parental PTS and youth PTSS falling between .65 and .73. The effect between parental CSE and youth CSE fell between .50 and .61 with 95% probability. Similarly, there was a 95% probability that the effect between parental communal coping and youth communal coping fell between .56 and .65. These findings suggested that a parent and a youth tend to have strong associations between the same variables.

### Summary of results

Results of the comparison between two Bayesian dyadic multilevel models favored the disaster distress model over the communal coping model. In the disaster distress model, lack of CSE mediated the relationship between communal coping and PTSS. For both parents and youths, CSE decreased as PTSS increased, which was associated with a decline in communal coping. Furthermore, results of the cross-pair correlations showed that an increase in youths’ communal coping was related to an increase in parents’ communal coping, and lower CSE in youths was related to lower CSE in parents. For both parents and youths, increased PTSS was associated with a greater increase in CSE when people had lower intercepts for lack of CSE compared to when they had higher intercepts for lack of CSE. Finally, the magnitude of the mediation effect of lack of CSE in the relationship between communal coping and PTSS was stronger for youths compared to parents.

## Discussion

CSE plays an important role in self-regulation when coping with trauma (Benight & Bandura, 2004). Our disaster distress model examined whether successful coping experiences increase CSE when children directly experience a traumatic event and overcome challenges related to trauma (i.e., gain mastery) (Bandura, 1997). Alternatively, our communal coping model tested whether children develop CSE through the observation of their parents who successfully cope with trauma or through verbal or nonverbal encouragement from their parents. We examined these processes in parent-youth dyads that experienced floods using Bayesian dyadic multilevel modeling. The disaster distress model included communal coping as a dependent variable, PTSS as an independent variable, and lack of CSE as a mediator variable. Alternatively, the communal coping model tested the effect of communal coping on PTSS with lack of CSE as a mediator. The results of the present study supported the disaster distress model over the communal coping model. Moreover, we found evidence for the mediation effect of lack of CSE in the relationship between PTSS and communal coping for both parents and youths. Our findings add to the theoretical understanding of the familial coping dynamics in parent-youth dyads.

Results of the present study showed that the disaster distress model is superior to the communal coping model. Previous disaster studies have found a similar mediation effect of CSE. Benight and Harper (2002) showed CSE mediated between acute disaster distress and subsequent posttraumatic distress a year later following a massive wildfire and flood disaster. In another longitudinal study following Hurricane Andrew, Benight et al. (1999a) showed CSE mediated the association between hurricane loss and general distress one-year later. In a cross-sectional study following Hurricane Opal, Benight et al. (1999b) showed CSE mediated the relationship between hurricane loss and distress as well as between social support and distress. Bosmans et al. (2013) demonstrated that CSE assessed 10 years after a massive fireworks factory disaster partially mediated between 4-year posttraumatic distress and 10-year posttraumatic distress.

These relationships are similar to the ones with social support in the social support deterioration model (Kaniasty & Norris, 1993; Norris & Kaniasty, 1996). This model suggests that post-trauma social support declines when the effect of disaster is severe, and this relationship leads to subsequent psychological distress. In the Benight et al. (1999b) Hurricane Opal cross-sectional study, CSE mediated the association between social support and posttraumatic distress suggesting that as social support declined, CSE also declined along with an increase in posttraumatic distress. Benight and Bandura (2004) argue that following a major traumatic event such as a disaster, one’s social resources are critical to promoting self-efficacious beliefs, thereby enhancing self-regulation and reducing distress.

In the context of CSE in the present study, people with higher levels of PTSS may struggle to self-regulate, which can result in a decline in their confidence in their ability to cope with challenges associated with the flood recovery (CSE decrease). Based on the present findings, the decrease in CSE related to managing PTSS has important implications for the family coping process. Our findings show that the lower CSE further leads to the decline in communal coping for both parents and youths. Indeed, Benight and Bandura (2004) suggested that lower CSE beliefs will result in poorer coping efforts, impairing effective goal pursuits and perseverance through recovery setbacks. Importantly, our study suggests this dynamic is seen in the family system.

For the first pathway between lack of CSE and PTSS for both parents and youths, the results of cross-pair correlations show that the correlations between lack of CSE and PTSS tend to be more positive when the intercept for lack of CSE is lower. These findings indicate that people with high CSE receive more negative impacts from PTSS. In other words, PTSS can chip away more portions of CSE when there are high amounts of CSE. This finding is consistent with Benight et al. (2017) where individuals with higher efficacy and higher PTSS following a motor vehicle crash showed a threshold negative shift in functioning over time.

The second pathway between communal coping and lack of CSE indicates that dyads that have diminished confidence in their coping abilities tend to cease further communal coping attempts after the flood disaster. The findings of the present study are consistent with previous research demonstrating that communal coping and CSE have a positive association (Helgeson et al., 2017; Van Vleet et al., 2019). It is possible that people who have high PTSS may interpret their symptoms as evidence of coping failure, driving less communal efforts due to an increase in ruminative self-focus. Indeed, negative life events have been linked to ruminative self-focus and increases in distress (Moberly & Watkins, 2008).

The results of the cross-pair correlations in the present study indicate that youths with high communal coping levels tend to have parents with high communal coping levels. Furthermore, youths with lower CSE levels have parents with lower CSE levels. It is plausible that parent’s CSE is bolstered when they observe the confidence in recovery from the disaster in their children, which can help reduce PTSS in parents. Indeed, our results show a relatively robust relationship between youth CSE and parental PTSS (95% credible interval [−.40, −.24]). However, we did not find any meaningful cross-pair intervariable correlations. Rather, the mediation effect of lack of CSE in the relationship between communal coping and PTSS is stronger for youths than for parents. These results indicate that youths’ CSE receives a severer hit when PTSS is higher, which leads to less frequent engagement into communal coping compared to parents. Youths tend to be affected more by traumatic experiences, and the symptoms can be more prolonged compared to adults (Clemmons et al., 2007; Cloitre et al., 2009). We reveal a possible psychological mechanism for youth vulnerability.

Because youths are more vulnerable in peri- and post-trauma (Clemmons et al., 2007; Cloitre et al., 2009), it is necessary to enhance resilience in youths to maintain their psychological health. Our model and other previous studies indicate that enhancing CSE is key to recovery post-disaster (Benight & Bandura, 2004). Results of the communal coping model suggest that communal coping can enhance CSE. Although we found that the disaster distress model is superior to the communal coping model, it is also a robust model on its own with successful model convergence and stable parameter estimates. Parents and guardians can purposefully communicate with their children about the disaster and make efforts to overcome challenges associated with the disaster as a family. In addition, parents and guardians can show their children that they cope well with disaster-related challenges. By observing their parents’ or guardians’ adjustment to new environments, youths can enhance their own CSE (Bandura, 1997). However, the literature on the effect of communal coping on CSE is still scarce, and more studies will be needed to fully understand the mechanism of this relationship.

It is crucial to provide external resources to sustain and maintain CSE post-disaster as Hobfoll et al. (2009) argued. Without external resources from the community, CSE becomes something similar to an empty hope, and CSE may diminish eventually. Previous studies consistently found that loss of resources is associated with lower CSE (Benight et al., 1999; Luszczynska et al., 2009). In addition to the direct effect of the loss of resources on CSE, the loss of resources can reduce perceived social support, which further reduces CSE (Warner et al., 2015). A pathway for how loss of resources affects parent-youth dynamics is still unclear, and further research will be needed.

### Limitations

Limitations of the present study included the use of a cross-sectional study design. A longitudinal study design would allow us to incorporate the chronological factor in the model and test how parent-youth dynamics change as time elapses. A related and more important issue is that we evaluated cross-sectional mediation models. Maxwell and Cole (2007) and Maxwell et al. (2011) demonstrated that a cross-sectional mediation analysis can be biased and misleading for testing a longitudinal mediation process. Although we agree with this perspective, there are situations where a cross-sectional mediation model can be informative as Shrout (2011) discussed. These circumstances include testing general indirect and direct effects rather than a longitudinal mediation process and testing models that are constructed based on well-founded theories. The strengths of our study included that our models are constructed based on well-founded theory, namely, social cognitive theory, and testing and comparing the models using a Bayesian analysis. Furthermore, we aimed to test models for a more general mechanism involving a potential indirect effect rather than a longitudinal mediation process. The timing of the assessments is critical in the longitudinal approach (Gollob & Reichardt, 1987). For example, elevated PTSS on a certain day leads to reduced CSE on the same day, but it might not be evident on CSE the next day or six months later. Our models depict the mediation effects occurring in a short temporal window (e.g., the same day).

Furthermore, we presented the simplest models possible that could test a dyadic relationship between parent and youths due to limited computational resources. Thus, we did not include some potentially important covariates such as geographical information (where participants lived), flood exposure levels, and socioeconomic status in the models. We have tested a model with the flood exposure levels as a random effects factor, but this model did not turn out to be a better model than Model 1, so we did not include it in the model presented in this study. However, both parents and youths with high flood exposure tend to have more mental health problems (Felix et al., 2019). This previous investigation indicates that a more complex model with the exposure variable as a fixed effects factor might reveal its effects on PTSS or communal coping. Furthermore, it should be noted that socioeconomic status can be an important factor associated with the recovery from a disaster (e.g., Fussell et al., 2010).

The diversity of our sample is also a limitation. The majority of our sample was identified as White. This percentage is greater than the proportion of people who identify as White in the total Texas population. Thus, the results of the present study need to be interpreted with caution when applying them to the overall Texas population or to the general population of the U.S. Similarly, more women participated as parents or guardians in this study. Thus, fathers’ data were less reflected in the sample. However, our Bayesian approach provides robust results with the 95% CIs and more predictive power compared to a non-Bayesian approach, so it is likely that coefficients based on more population-representative data would fall within the 95% CIs found in this study. Finally, the measures for communal coping and CSE-T have not been validated for children and adolescents; thus, it is possible that these measures can have different factor structures from an adult sample. However, it should be noted that internal consistency for these measures is robust.

Our study is the first disaster-focused investigation to compare two competing models involving CSE as a mediator in the relationship between communal coping and PTSS using Bayesian dyadic multilevel modeling. We found that the disaster distress model was a favorable model over the communal coping model. Results of the disaster distress model demonstrate that CSE declines as PTSS gets more severe, which further leads to decreased communal coping for both parents and youths. This mediation effect of CSE in the relationship between PTSS and communal coping is stronger for youths compared to parents, indicating that children’s CSE is affected more by severe PTSS. Although our study has limitations, our unique approach using Bayesian dyadic multilevel modeling offers a theoretical understanding of the familial coping dynamics following a major disaster and how families and youths develop CSE. Lastly, our findings will be useful for future studies as our results can be directly incorporated into future Bayesian analyses as priors.

## Supplementary Material

1

2

3

4

## Figures and Tables

**Table 1.** Demographics for the sample and means and standard deviation for the study variables

		Parents		Youths	
			
Variable	Level	Count	%	Count	%

Gender	Female	334	69.0	228	47.2

	Male	150	31.0	255	52.8

Relationship to youths	Mother	319	66.3		

	Father	125	26.0		

	Stepmother	14	2.9		

	Stepfather	5	1.0		

	Grandparent	6	1.2		

	Guardian	9	1.9		

	Aunt/uncle	3	0.6		

Ethnicity	Native American	7	1.5	4	0.9

	Asian/Pacific Islander	37	7.7	32	7.2

	Black/African American	44	9.2	41	9.2

	White	298	62.2	255	57.3

	Hispanic/Latino	87	18.2	87	19.6

	Mixed	6	1.3	25	5.6

Education	Elementary school	2	0.4		

	Junior high school	4	0.8		

	High school	75	15.5		

	Vocational/trade school	24	5.0		

	Community college	28	5.8		

	Some college	124	25.6		

	Four-year college	145	30.0		

	Graduate school	82	16.9		

*Note. N* = 485.

**Table 2.** Informative priors for the disaster distress hypothesis and communal coping hypothesis models

Model	DV	Class	Prior	Reason
Disaster Distress				
	Communal coping			
		Coefficient for coping self-efficacy	*N* (.21, .1)	Mean r coefficient and its SD from Helgeson et al., (2017) and Van Vleet et al. (2019)
		Coefficient for posttraumatic stress symptoms	Student *t* (3, 0, 1)	Not enough prior studies. Weakly informative priors recommended by Gelman (2020).
		Intercept	Student *t* (3, 0, 1)	Weakly informative priors recommended by Gelman (2020).
		SD	Student *t* (3, 0, 1)	Weakly informative priors recommended by Gelman (2020).
		Shape	Gamma (1, 1)	Visual inspection of the communal coping distribution generally followed a Gamma distribution (*α* = 1, rate = 1).
	Coping self-efficacy			
		Coefficient for posttraumatic stress symptoms	*N* (.36, .06)	Weighted r between self-efficacy and symptom severity from a meta-analysis (Luszczynska et al., 2009)
		Intercept	Student *t* (3, 0, 1)	Weakly informative priors recommended by Gelman (2020).
		SD	Student *t* (3, 0, 1)	Weakly informative priors recommended by Gelman (2020).
		Shape	Gamma (1, 1)	Visual inspection of the reverse-coded CSE distribution generally followed a Gamma distribution (*α* = 1, rate = 1).
Communal Coping				
	Posttraumatic stress symptoms			
		Coefficient for coping self-efficacy	*N* (.36, .06)	Weighted r between self-efficacy and symptom severity from a meta-analysis (Luszczynska et al., 2009)
		Coefficient for communal coping	Student *t* (3, 0, 1)	Not enough prior studies. Weakly informative prior recommended by Gelman (2020).
		Intercept	Student *t* (3, 0, 1)	Weakly informative priors recommended by Gelman (2020).
		SD	Student *t* (3, 0, 1)	Weakly informative priors recommended by Gelman (2020).
		Shape	Gamma (1, 1)	Visual inspection of the PTS distribution generally followed a Gamma distribution (*α* = 1, rate = 1).
	Coping self-efficacy			
		Coefficient for communal coping	*N* (.21, .1)	Mean r coefficient and its SD from Helgeson et al. (2017) and Van Vleet et al. (2019)
		Intercept	Student *t* (3, 0, 1)	Weakly informative priors recommended by Gelman (2020).
		SD	Student *t* (3, 0, 1)	Weakly informative priors recommended by Gelman (2020).
		Shape	Gamma (1, 1)	Visual inspection of the reverse-coded CSE distribution generally followed a Gamma distribution (*α* = 1, rate = 1).

*Note*. The same priors were applied for both parents and youths. *N* = normal distribution; Student *t* = student *t* distribution; Gamma = gamma distribution; SD = standard deviation.

**Table 3.** Central tendency and 95% credible intervals for coefficients, $\bar{R}$, bulk ESS, and tail ESS for the Bayesian dyadic multilevel modeling (disaster distress model)

Sub-component	IV	β	95% CI	$\bar{R}$	Bulk ESS	Tail ESS
Parent (DV = Communal coping)						
	Intercept	.13	.01, .25	1.00	25,045	28,181
	Posttraumatic stress symptoms	.04	−.02, .10	1.00	27,238	29,107
	Lack of coping self-efficacy	.12	.06, .17	1.00	29,749	30,092
Parent (DV = Lack of coping self-efficacy)						
	Intercept	−.06	−.21, .08	1.00	20,191	26,041
	Posttraumatic stress symptoms	.25	.19, .32	1.00	26,241	27,996
Youth (DV = Communal coping)						
	Intercept	.14	.02, .26	1.00	27,214	28,316
	Posttraumatic stress symptoms	−.03	−.09, .03	1.00	29,237	29,270
	Lack of coping self-efficacy	.18	.13, .23	1.00	28,402	28,745
Youth (DV = Lack of coping self-efficacy)						
	Intercept	−.02	−.16, .13	1.00	15,108	24,038
	Posttraumatic stress symptoms	.27	.20, .34	1.00	21,720	27,631

**Table 4.** Central tendency and 95% credible intervals for coefficients, $\bar{R}$, bulk ESS, and tail ESS for the Bayesian dyadic multilevel modeling (communal coping model)

Sub-component	IV	β	95% CI	$\bar{R}$	Bulk ESS	Tail ESS
Parent (DV = Posttraumatic stress symptoms)						
	Intercept	−.10	−.24, .04	1.00	22,774	27,528
	Communal coping	.03	−.03, .10	1.00	28,512	28,890
	Lack of coping self-efficacy	.24	.18, .31	1.00	25,777	29,096
Parent (DV = Lack of coping self-efficacy)						
	Intercept	.12	−.03, .26	1.00	17,311	23,442
	Communal coping	.18	.12, .25	1.00	26,254	29,468
Youth (DV = Posttraumatic stress symptoms)						
	Intercept	−.33	−.50, −.15	1.00	24,396	28,323
	Communal coping	.03	−.05, .11	1.00	28,642	29,500
	Lack of coping self-efficacy	.26	.19, .33	1.00	28,449	29,383
Youth (DV = Lack of coping self-efficacy)						
	Intercept	.06	−.08, .20	1.00	21,656	27,201
	Communal coping	.22	.16, .29	1.00	27,712	28,111

**Table 5.** Intercorrelations of the parameters and cross-pair correlations of the parameters in the Bayesian dyadic multilevel modeling for disaster distress model

Parameter	Beta	95% CIs	$\bar{R}$	Bulk ESS	Tail ESS
**Parent CCOPE Intercept, Youth CCOPE Intercept**	.87	.70, .97	1.00	11,325	17,791
Parent CCOPE Intercept, Parent CCOPE-Parent PTSS	−.27	−.80, .51	1.00	22,680	27,074
Parent CCOPE Intercept, Parent CCOPE-Parent Lack of CSE	−.07	−.64, .62	1.00	16,947	26,427
Parent CCOPE Intercept, Youth CCOPE-Youth PTSS	.09	−.62, .72	1.00	27,115	28,238
Parent CCOPE Intercept, Youth CCOPE-Youth Lack of CSE	−.21	−.78, .53	1.00	26,015	28,195
Youth CCOPE Intercept, Parent CCOPE-Parent PTSS	−.17	−.75, .55	1.00	23,016	26,460
Youth CCOPE Intercept, Parent CCOPE-Parent Lack of CSE	.08	−.56, .69	1.00	24,561	24,437
Youth CCOPE Intercept, Youth CCOPE-Youth PTSS	.01	−.64, .68	1.00	27,367	29,238
Youth CCOPE Intercept, Youth CCOPE-Youth Lack of CSE	−.34	−.83, .47	1.00	24,618	28,159
**Parent Lack of CSE Intercept, Youth Lack of CSE Intercept**	.48	.25, .70	1.00	10,993	17,072
**Parent Lack of CSE Intercept, Parent Lack of CSE-Parent PTS**	−.89	−.96, −.79	1.00	9814	15,822
Parent Lack of CSE Intercept, Youth Lack of CSE-Youth PTSS	−.38	−.76, .03	1.00	9287	14,851
Youth Lack of CSE Intercept, Parent Lack of CSE-Parent PTSS	−.24	−.61, .14	1.00	6382	9637
**Youth Lack of CSE Intercept, Youth Lack of CSE-Youth PTSS**	−.86	−.95, −.69	1.00	12,586	16,743
Parent CCOPE-Parent PTSS, Parent CCOPE-Parent Lack of CSE	−.05	−.73, .67	1.00	16,405	24,578
Parent CCOPE-Parent PTSS, Youth CCOPE-Youth PTS	.05	−.68, .74	1.00	26,861	29,048
Parent CCOPE-Parent Lack of CSE, Youth CCOPE-Youth PTSS	.01	−.70, .72	1.00	29,566	29,519
Parent CCOPE-Parent PTSS, Youth CCOPE-Youth Lack of CSE	.05	−.68, .74	1.00	27,051	28,407
Parent CCOPE-Parent Lack of CSE, Youth CCOPE-Youth Lack of CSE	.05	−.68, .74	1.00	24,442	28,939
Youth CCOPE-Youth PTSS, Youth CCOPE-Youth Lack of CSE	−.07	−.75, .67	1.00	27,223	29,048
Parent Lack of CSE-Parent PTSS, Youth Lack of CSE-Youth PTSS	.34	−.15, .70	1.00	9325	14,623

*Note*. CCOPE = communal coping; PTSS = posttraumatic symptoms; CSE = trauma coping self-efficacy; CI = credible interval. In the Parameter column, the notion, *X-Y*, indicates the association between variable *X* and variable *Y*. The parameter in bold face indicated that its 95% CIs do not include 0.

**Table 6.** Central tendency and 95% credible intervals for coefficients, $\bar{R}$, bulk ESS, and tail ESS for the Bayesian dyadic multilevel modeling for the model with weakly informative priors in the sensitivity analysis

Sub-model	IV	Beta	95% CI	$\bar{R}$	Bulk ESS	Tail ESS
Parent (DV = Communal coping)						
	Intercept	.14	.01, .26	1.00	25,262	28,853
	Posttraumatic stress symptoms	.04	−.02, .10	1.00	28,852	29,672
	Lack of coping self-efficacy	.11	.05, .16	1.00	28,872	28,700
Parent (DV = Lack of coping self-efficacy)						
	Intercept	.04	−.12, .21	1.00	19,896	27,538
	Posttraumatic stress symptoms	.19	.11, .27	1.00	25,254	28,862
Youth (DV = Communal coping)						
	Intercept	.14	.03, .26	1.00	26,163	28,973
	Posttraumatic stress symptoms	−.03	−.09, .04	1.00	28,997	29,231
	Lack of coping self-efficacy	.18	.12, .23	1.00	28,102	29,397
Youth (DV = Lack of coping self-efficacy)						
	Intercept	.07	−.09, .24	1.00	13,088	20,838
	Posttraumatic stress symptoms	.21	.13, .29	1.00	19,024	26,134

**Table 7.** Ninety-five percent credible intervals and point estimates of bayesian correlations, means, and standard deviations for the study variables

	1	2	3	4	5	6	7
1. PPTSS	1.38 (1.09)						
2. YPTS	.69 [.65, .73]	1.11 (1.10)					
3. PCSE	−.27 [−.35, −.19]	−.16 [−.25, −.08]	5.60 (1.22)				
4. YCSE	−.32 [−.40, −.24]	−.27 [−.35, −.18]	.56 [.50, .61]	5.64 (1.22)			
5. PCC	−.14 [−.22, −.05]	−.10 [−.19, −.01]	.28 [.20, .36]	.33 [.25, .41]	5.58 (1.13)		
6. YCC	−.09 [−.18, −.00]	−.06 [−.14, .04]	.24 [.15, .32]	.41 [.33, .48]	.61 [.55, .65]	5.56 (1.18)	
7. Time	.01 [−.08, .10]	.03 [−.06, .10]	−.01 [−.10, .08]	−.04 [−.13, .05]	−.01 [−.11, .08]	.03 [−.06, .12]	416.75 (160.78)

*Note*. The values in the lower half below the diagonal line indicate the point estimates and 95% credible intervals in the brackets [lower limit, higher limit]; The values in the diagonal line indicate means and standard deviations in the parentheses. PPTSS = parental posttraumatic stress symptoms; PCSE = parental trauma coping self-efficacy; PCC = parental communal coping; YPTSS = youth posttraumatic stress symptoms; YCSE = youth trauma coping self-efficacy; YCC = youth communal coping. Time: time elapsed since the disasters in days. The 95% credible intervals indicate that 95% of all possible values fall within this range.
