# Supplementary material for: Coping self-efficacy mediates effects of posttraumatic distress on communal coping in parent-adolescence dyads after floods
Source: Dev Psychopathol. Author manuscript; Available in PMC 2025 Nov 1. (PMC11401968; doi:10.1017/S0954579424000567)
Supplement: 1 [file NIHMS1968016-supplement-1.pdf]

```
#================================bayes==========================================
library(brms)
library(shinystan)
library(bayesplot)
library(tidybayes)
library(ggmcmc)

##=======================model1: disaster distress model======================
#pccopeR = parent communal coping (reverse coded); ppts = parent posttraumatic
stress symptoms;
#pcseR = parent coping self-efficacy (reverse coded); id = subject term;
#cccopeR = youth coping self-efficacy (reverse coded); cpts = youth posttraumatic
stress symptoms;
#ccseR = youth coping self-efficacy (reverse coded);


###parents
bfpccope = bf(pccopeR ~ ppts + pcseR + (1 + ppts + pcseR|a|id))
bfpcse = bf(pcseR ~ ppts + (1 + ppts|b|id))

###children
bfcccope = bf(cccopeR ~ cpts + ccseR + (1 + cpts + ccseR|a|id))
bfccse = bf(ccseR ~ cpts + (1 + cpts|b|id))

###fit model1
get_prior(bfpccope + bfpcse + bfcccope + bfccse + set_rescor(F), data=data,
          family=hurdle_gamma(), control=list(adapt_delta=.9, max_treedepth=15))

priors = c(set_prior('normal(.21,.1)', class='b', coef='ccseR', resp='cccopeR'),
           set_prior('normal(.2,.2)', class='b', coef='cpts', resp='cccopeR'),
           set_prior('student_t(3,0,1)', class='Intercept', resp='cccopeR'),
           set_prior('student_t(3,0,1)', class='sd', resp='cccopeR'),
           set_prior('gamma(1,1)', class='shape', resp='cccopeR'),
           set_prior('normal(.36,.06)', class='b', coef='cpts', resp='ccseR'),
           set_prior('student_t(3,0,1)', class='Intercept', resp='ccseR'),
           set_prior('student_t(3,0,1)', class='sd', resp='ccseR'),
           set_prior('gamma(1,1)', class='shape', resp='ccseR'),
           set_prior('normal(.2,.2)', class='b', coef='ppts', resp='pccopeR'),
           set_prior('normal(.21,.1)', class='b', coef='pcseR', resp='pccopeR'),
           set_prior('student_t(3,0,1)', class='Intercept', resp='pccopeR'),
           set_prior('student_t(3,0,1)', class='sd', resp='pccopeR'),
           set_prior('gamma(1,1)', class='shape', resp='pccopeR'),
           set_prior('normal(.36,.06)', class='b', coef='ppts', resp='pcseR'),
           set_prior('student_t(3,0,1)', class='Intercept', resp='pcseR'),
           set_prior('student_t(3,0,1)', class='sd', resp='pcseR'),
           set_prior('gamma(1,1)', class='shape', resp='pcseR'))

mod1_1 = brm(bfpccope + bfpcse + bfcccope + bfccse + set_rescor(F), data=data,
           iter=7500, chains=32, warmup=3750, seed=102, thin=4, autocor=NULL,
           normalize=T, cores=32, prior=priors, init=0, family=hurdle_gamma(),
```

```
            control=list(adapt_delta=.999, max_treedepth=15),
            save_pars=save_pars(all=T))

summary(mod1_1)

plot(mod1_1)

a = pp_check(mod1_1, resp='pccopeR', ndraws=500)
a + ggtitle('Parent Communal Coping') + xlim(0, 7.5)
b = pp_check(mod1_1, resp='cccopeR', ndraws=500)
b + ggtitle('Youth Communal Coping') + xlim(0, 7.5)
c = pp_check(mod1_1, resp='pcseR', ndraws=500)
c + ggtitle('Parent Coping Self-Efficacy') + xlim(0, 7.5)
d = pp_check(mod1_1, resp='ccseR', ndraws=500)
d + ggtitle('Youth Coping Self-Efficacy') + xlim(0, 7.5)

##====================model2: communal coping model===========================

###parents
bfppts = bf(ppts ~ pccopeR + pcseR + (1 + pccopeR + pcseR|a|id))
bfpcse = bf(pcseR ~ pccopeR + (1 + pccopeR|b|id))

###children
bfcpts = bf(cpts ~ cccopeR + ccseR + (1 + cccopeR + ccseR|a|id))
bfccse = bf(ccseR ~ cccopeR + (1 + cccopeR|b|id))

###fit model2

get_prior(bfppts + bfpcse + bfcpts + bfccse + set_rescor(F), data=data,
          iter=1000, chains=4, warmup=250, cores=10, normalize=T, seed=103
          control=list(adapt_delta=.9, max_treedepth=15))

priors = c(set_prior('normal(.21,.1)', class='b', coef='cccopeR', resp='ccseR'),
           set_prior('student_t(3,0,1)', class='Intercept', resp='ccseR'),
           set_prior('student_t(3,0,1)', class='sd', resp='ccseR'),
           set_prior('gamma(1,1)', class='shape', resp='ccseR'),
           set_prior('normal(.2,.2)', class='b', coef='cccopeR', resp='cpts'),
           set_prior('normal(.36,.06)', class='b', coef='ccseR', resp='cpts'),
           set_prior('student_t(3,0,1)', class='Intercept', resp='cpts'),
           set_prior('student_t(3,0,1)', class='sd', resp='cpts'),
           set_prior('gamma(1,1)', class='shape', resp='cpts'),
           set_prior('normal(.21,.1)', class='b', coef='pccopeR', resp='pcseR'),
           set_prior('student_t(3,0,1)', class='Intercept', resp='pcseR'),
           set_prior('student_t(3,0,1)', class='sd', resp='pcseR'),
           set_prior('gamma(1,1)', class='shape', resp='pcseR'),
           set_prior('normal(.36,.06)', class='b', coef='pcseR', resp='ppts'),
           set_prior('normal(.2,.2)', class='b', coef='pccopeR', resp='ppts'),
           set_prior('student_t(3,0,1)', class='Intercept', resp='ppts'),
           set_prior('student_t(3,0,1)', class='sd', resp='ppts'),
           set_prior('gamma(1,1)', class='shape', resp='ppts'))
```

```
mod2_1 = brm(bfppts + bfpcse + bfcpts + bfccse + set_rescor(F), data=data,
           iter=7500, chains=32, warmup=3750, normalize=T, seed=103, cores=32,
           control=list(adapt_delta=.999, max_treedepth=15), prior=priors,
           init=0, family=hurdle_gamma(), autocor=NULL, thin=4,
           save_pars=save_pars(all=T))

summary(mod2_1)

plot(mod2_1)

e = pp_check(mod2_1, resp='ppts', ndraws=500)
e + ggtitle('Parent Posttraumatic Stress Symptoms') + xlim(0, 6)
f = pp_check(mod2_1, resp='cpts', ndraws=500)
f + ggtitle('Youth Posttraumatic Stress Symptoms') + xlim(0, 6)
g = pp_check(mod2_1, resp='pcseR', ndraws=500)
g + ggtitle('Parent Coping Self-Efficacy') + xlim(0, 7.5)
h = pp_check(mod2_1, resp='ccseR', ndraws=500)
h + ggtitle('Youth Coping Self-Efficacy') + xlim(0, 7.5)

##==============weakly informative priors: disaster distress model==============

###parents
bfpccope = bf(pccopeR ~ ppts + pcseR + (1 + ppts + pcseR|a|id))
bfpcse = bf(pcseR ~ ppts + (1 + ppts|b|id))

###children
bfcccope = bf(cccopeR ~ cpts + ccseR + Time_Dis + (1 + cpts + ccseR|a|id))
bfccse = bf(ccseR ~ cpts + (1 + cpts|b|id))

###fit
get_prior(bfpccope + bfpcse + bfcccope + bfccse + set_rescor(F), data=data,
          family=hurdle_gamma(), iter=7500, chains=32, warmup=3750, seed=102,
          normalize=T, cores=32, prior=priors,
          control=list(adapt_delta=.999, max_treedepth=15))

priors = c(set_prior('student_t(3,0,1)', class='b', coef='ccseR', resp='cccopeR'),
           set_prior('student_t(3,0,1)', class='b', coef='cpts', resp='cccopeR'),
           set_prior('student_t(3,0,1)', class='Intercept', resp='cccopeR'),
           set_prior('student_t(3,0,1)', class='sd', resp='cccopeR'),
           set_prior('gamma(1,1)', class='shape', resp='cccopeR'),
           set_prior('student_t(3,0,1)', class='b', coef='cpts', resp='ccseR'),
           set_prior('student_t(3,0,1)', class='Intercept', resp='ccseR'),
           set_prior('student_t(3,0,1)', class='sd', resp='ccseR'),
           set_prior('gamma(1,1)', class='shape', resp='ccseR'),
           set_prior('student_t(3,0,1)', class='b', coef='ppts', resp='pccopeR'),
           set_prior('student_t(3,0,1)', class='b', coef='pcseR', resp='pccopeR'),
           set_prior('student_t(3,0,1)', class='Intercept', resp='pccopeR'),
           set_prior('student_t(3,0,1)', class='sd', resp='pccopeR'),
           set_prior('gamma(1,1)', class='shape', resp='pccopeR'),
           set_prior('student_t(3,0,1)', class='b', coef='ppts', resp='pcseR'),
           set_prior('student_t(3,0,1)', class='Intercept', resp='pcseR'),
```

```
           set_prior('student_t(3,0,1)', class='sd', resp='pcseR'),
           set_prior('gamma(1,1)', class='shape', resp='pcseR'))

mod1_2 = brm(bfpccope + bfpcse + bfcccope + bfccse + set_rescor(F), data=data,
           family=hurdle_gamma(), iter=7500, chains=32, warmup=3750, seed=104,
           normalize=T, cores=32, prior=priors, init=0,
           control=list(adapt_delta=.999, max_treedepth=15), thin=4,
           save_pars=save_pars(all=T))

summary(mod1_2)

plot(mod1_2)

a = pp_check(mod1_2, resp='pccopeR', ndraws=500)
a + ggtitle('Parent Communal Coping') + xlim(0, 7.5)
b = pp_check(mod1_2, resp='cccopeR', ndraws=500)
b + ggtitle('Youth Communal Coping') + xlim(0, 7.5)
c = pp_check(mod1_2, resp='pcseR', ndraws=500)
c + ggtitle('Parent Coping Self-Efficacy') + xlim(0, 7.5)
d = pp_check(mod1_2, resp='ccseR', ndraws=500)
d + ggtitle('Youth Coping Self-Efficacy') + xlim(0, 7.5)

##==========================model comparison========================================
B_factor = brms::bayes_factor(mod1_1, mod2_1)

B_factor2 = brms::bayes_factor(mod1_1, mod1_2)

##===========================test hypothesis========================================
library(ggplot2)

h = c('pcseR_ppts*pccopeR_pcseR > 0.01', 'pcseR_ppts*pccopeR_pcseR > 0.0125',
      'pcseR_ppts*pccopeR_pcseR > 0.015', 'pcseR_ppts*pccopeR_pcseR > 0.0175',
      'pcseR_ppts*pccopeR_pcseR > 0.02', 'pcseR_ppts*pccopeR_pcseR > 0.0225',
      'pcseR_ppts*pccopeR_pcseR > 0.025', 'pcseR_ppts*pccopeR_pcseR > 0.0275',
      'pcseR_ppts*pccopeR_pcseR > 0.03', 'pcseR_ppts*pccopeR_pcseR > 0.0325',
      'pcseR_ppts*pccopeR_pcseR > 0.035', 'pcseR_ppts*pccopeR_pcseR > 0.0375',
      'pcseR_ppts*pccopeR_pcseR > 0.04', 'pcseR_ppts*pccopeR_pcseR > 0.0425',
      'pcseR_ppts*pccopeR_pcseR > 0.045', 'pcseR_ppts*pccopeR_pcseR > 0.0475',
      'pcseR_ppts*pccopeR_pcseR > 0.05', 'pcseR_ppts*pccopeR_pcseR > 0.0525')
h1 = hypothesis(mod1_1, h)
p_p = as.data.frame(h1$hypothesis$Post.Prob)
p_p$Beta = c(.010, .0125, .015, .0175, .020, .0225, .025, .0275, .030, .0325,
             .035, .0375, .040, .0425, .045, .0475, .050, .0525)

ggplot(p_p, aes(y=h1$hypothesis$Post.Prob, x=Beta)) +
          geom_line() +
          labs(x='Beta Coefficient', y='Posterior Probability') +
          ggtitle('Parents')


h = c('ccseR_cpts*cccopeR_ccseR > 0.01', 'ccseR_cpts*cccopeR_ccseR > 0.0125',
```

```
      'ccseR_cpts*cccopeR_ccseR > 0.015', 'ccseR_cpts*cccopeR_ccseR > 0.0175',
      'ccseR_cpts*cccopeR_ccseR > 0.02', 'ccseR_cpts*cccopeR_ccseR > 0.0225',
      'ccseR_cpts*cccopeR_ccseR > 0.025', 'ccseR_cpts*cccopeR_ccseR > 0.0275',
      'ccseR_cpts*cccopeR_ccseR > 0.03', 'ccseR_cpts*cccopeR_ccseR > 0.0325',
      'ccseR_cpts*cccopeR_ccseR > 0.035', 'ccseR_cpts*cccopeR_ccseR > 0.0375',
      'ccseR_cpts*cccopeR_ccseR > 0.04', 'ccseR_cpts*cccopeR_ccseR > 0.0425',
      'ccseR_cpts*cccopeR_ccseR > 0.045', 'ccseR_cpts*cccopeR_ccseR > 0.0475',
      'ccseR_cpts*cccopeR_ccseR > 0.05', 'ccseR_cpts*cccopeR_ccseR > 0.0525',
      'ccseR_cpts*cccopeR_ccseR > 0.055', 'ccseR_cpts*cccopeR_ccseR > 0.0575',
      'ccseR_cpts*cccopeR_ccseR > 0.06', 'ccseR_cpts*cccopeR_ccseR > 0.0625',
      'ccseR_cpts*cccopeR_ccseR > 0.065', 'ccseR_cpts*cccopeR_ccseR > 0.0675',
      'ccseR_cpts*cccopeR_ccseR > 0.07', 'ccseR_cpts*cccopeR_ccseR > 0.0725')
h2 = hypothesis(mod1_1, h)
p_p = as.data.frame(h2$hypothesis$Post.Prob)
p_p$Beta = c(.010, .0125, .015, .0175, .020, .0225, .025, .0275, .030, .0325,
             .035, .0375, .040, .0425, .045, .0475, .050, .0525, .055, .0575,
             .060, .0625, .065, .0675, .070, .0725)

ggplot(p_p, aes(y=h2$hypothesis$Post.Prob, x=Beta)) +
           geom_line() +
           labs(x='Beta Coefficient', y='Posterior Probability') +
           ggtitle('Youths')

#================================bayes correlation================================
data <-
  data %>%
  mutate(ppts_s = scale(ppts),
         cpts_s = scale(cpts),
         pcse_s = scale(pcse),
         ccse_s = scale(ccse),
         pccope_s = scale(pccope),
         cccope_s = scale(cccope))

get_prior(bf(mvbind(ppts_s, cpts_s, pcse_s, ccse_s, pccope_s, cccope_s)~0,
             sigma~0) + set_rescor(T), data=data, chains=4, cores=4, seed=110,
          family=gaussian)

priors = set_prior('lkj(1)', class='rescor')

b_cor = brm(bf(mvbind(ppts_s, cpts_s, pcse_s, ccse_s, pccope_s, cccope_s)~0,
            sigma~0) + set_rescor(T), data=data, chains=4, cores=4, seed=110,
            family=gaussian, prior=priors)

summary(b_cor)
plot(b_cor)
posterior_summary(b_cor)
mcmc_plot(b_cor, type='dens', fixed=T)
```
