# Supplementary figures and images for: Coping self-efficacy mediates effects of posttraumatic distress on communal coping in parent-adolescence dyads after floods
Source: Dev Psychopathol. Author manuscript; Available in PMC 2025 Nov 1. (PMC11401968; doi:10.1017/S0954579424000567)

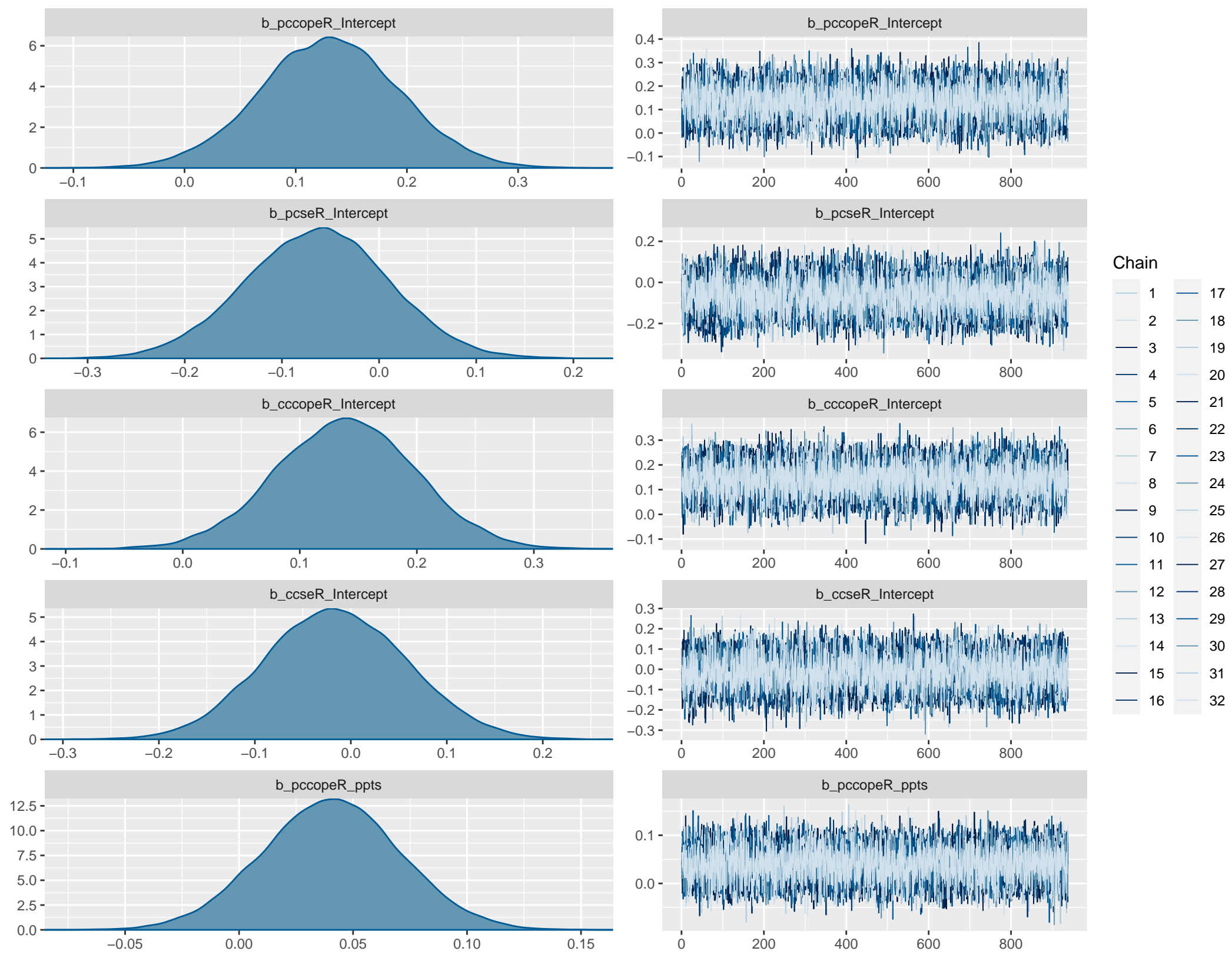

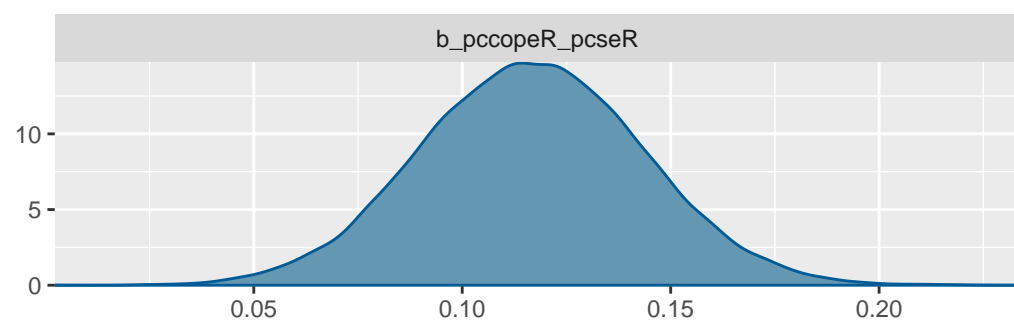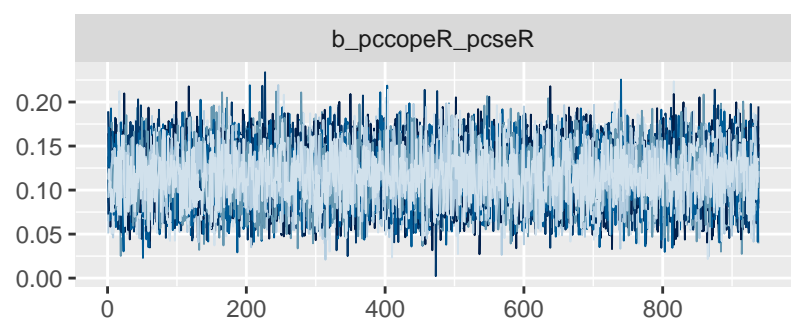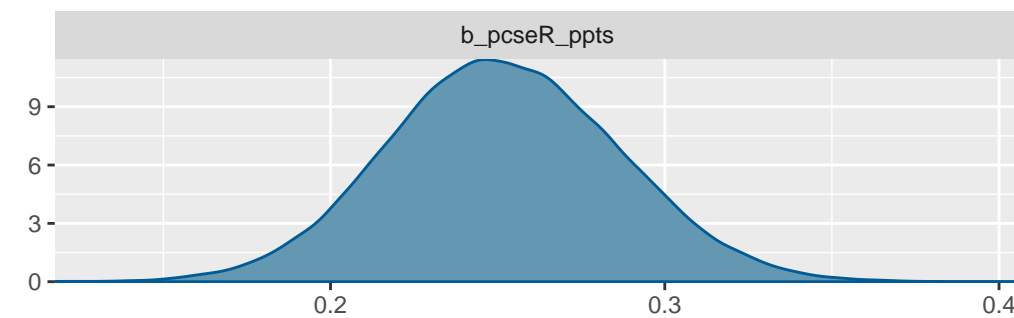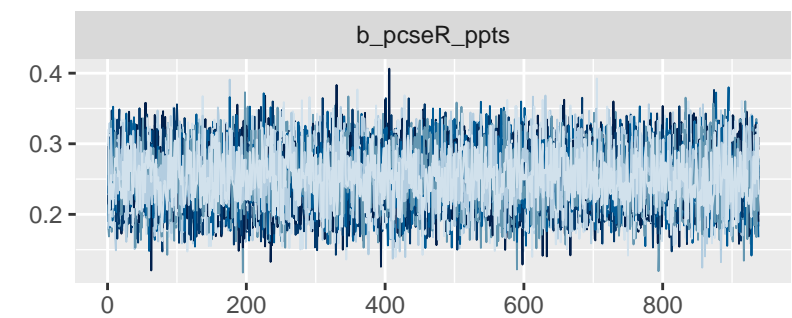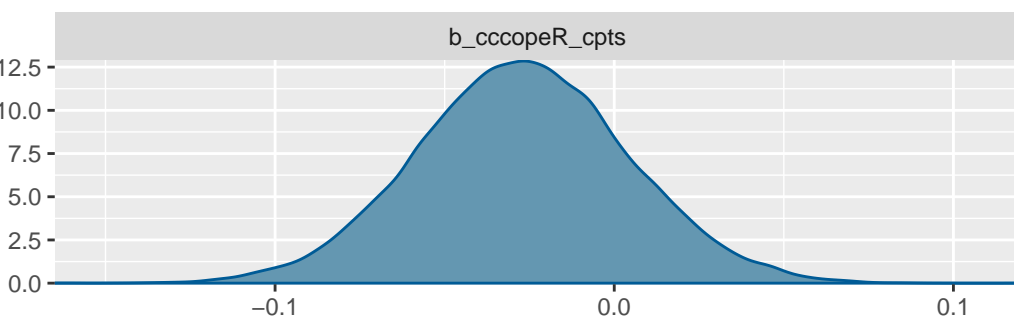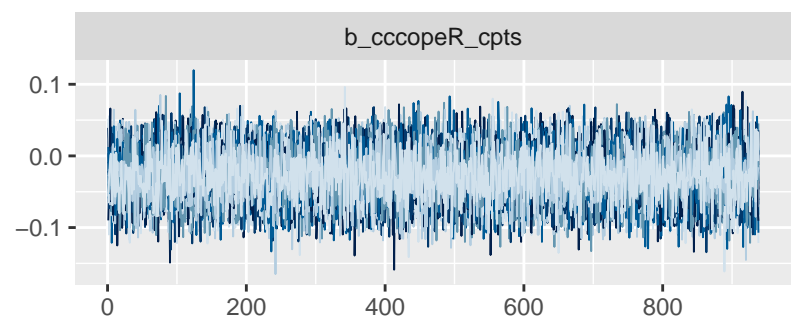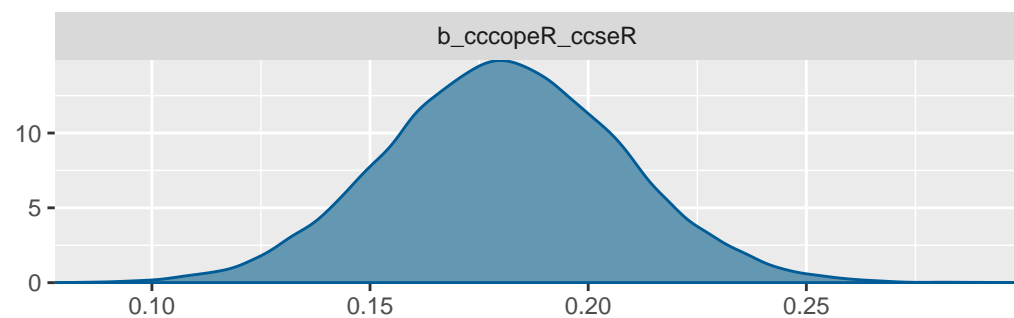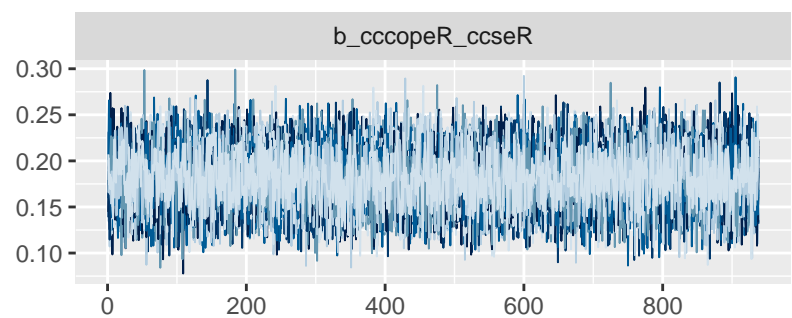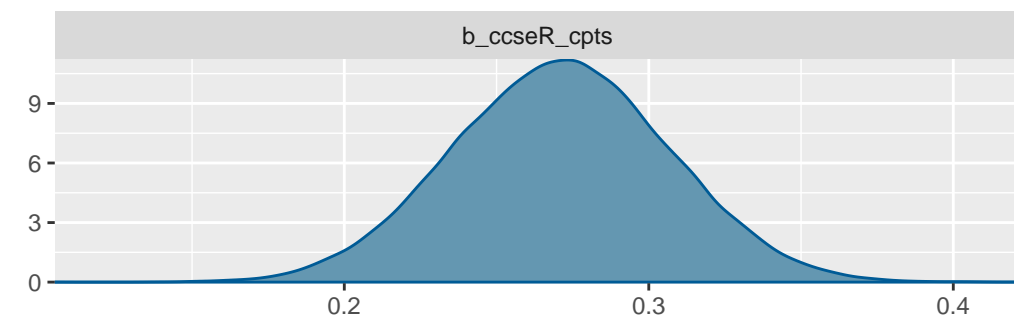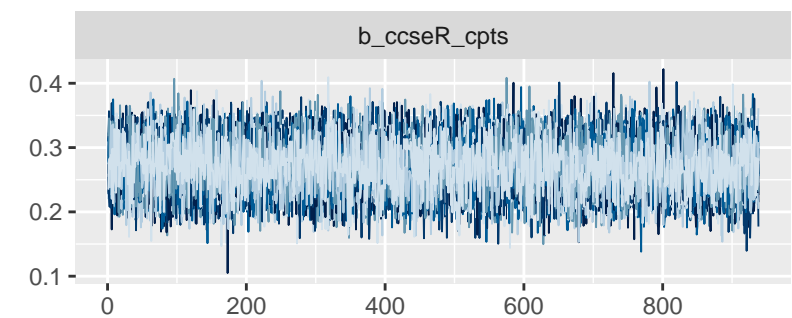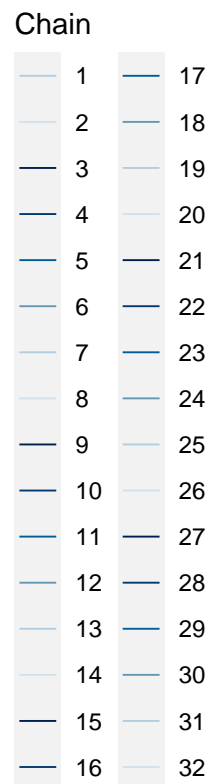

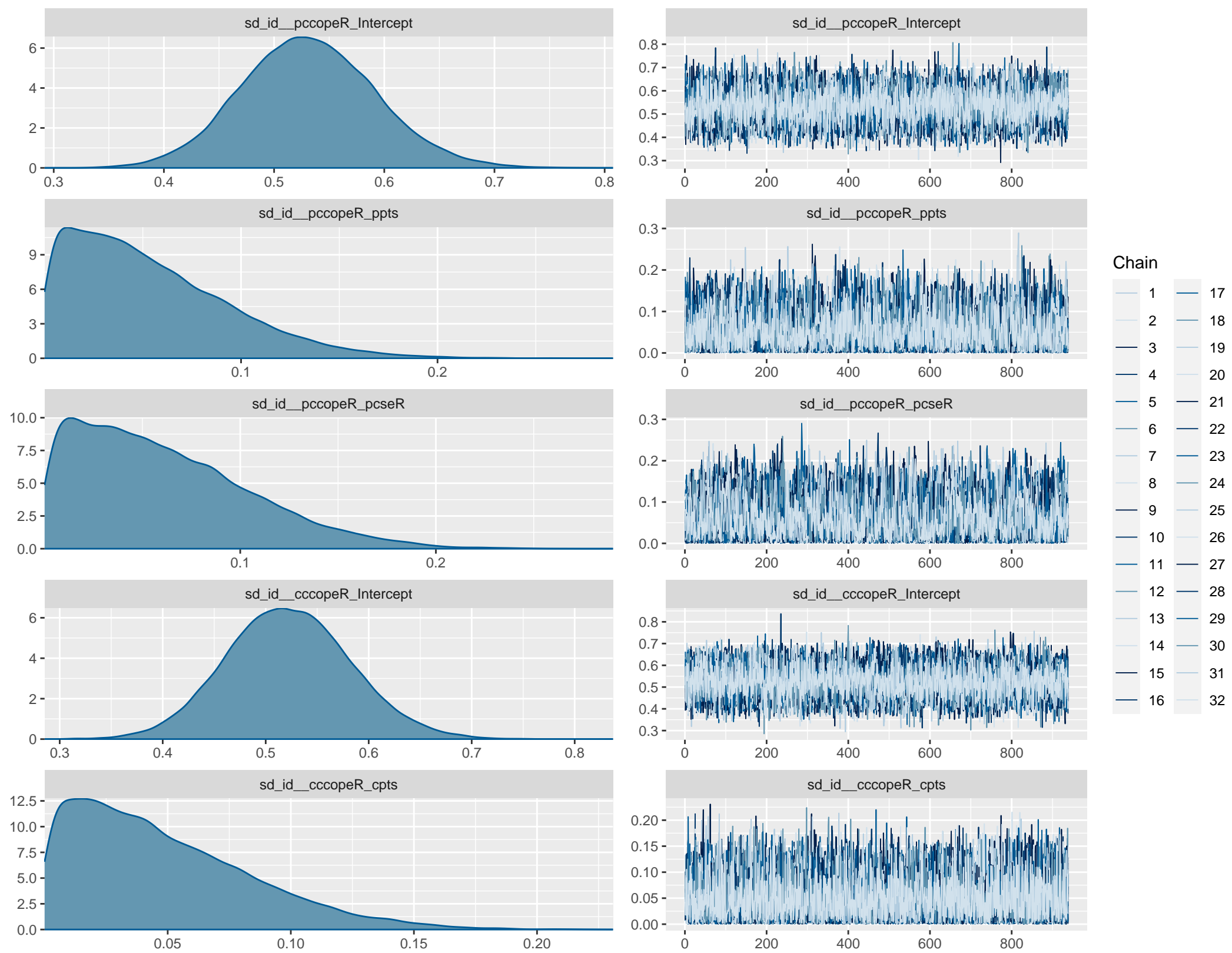

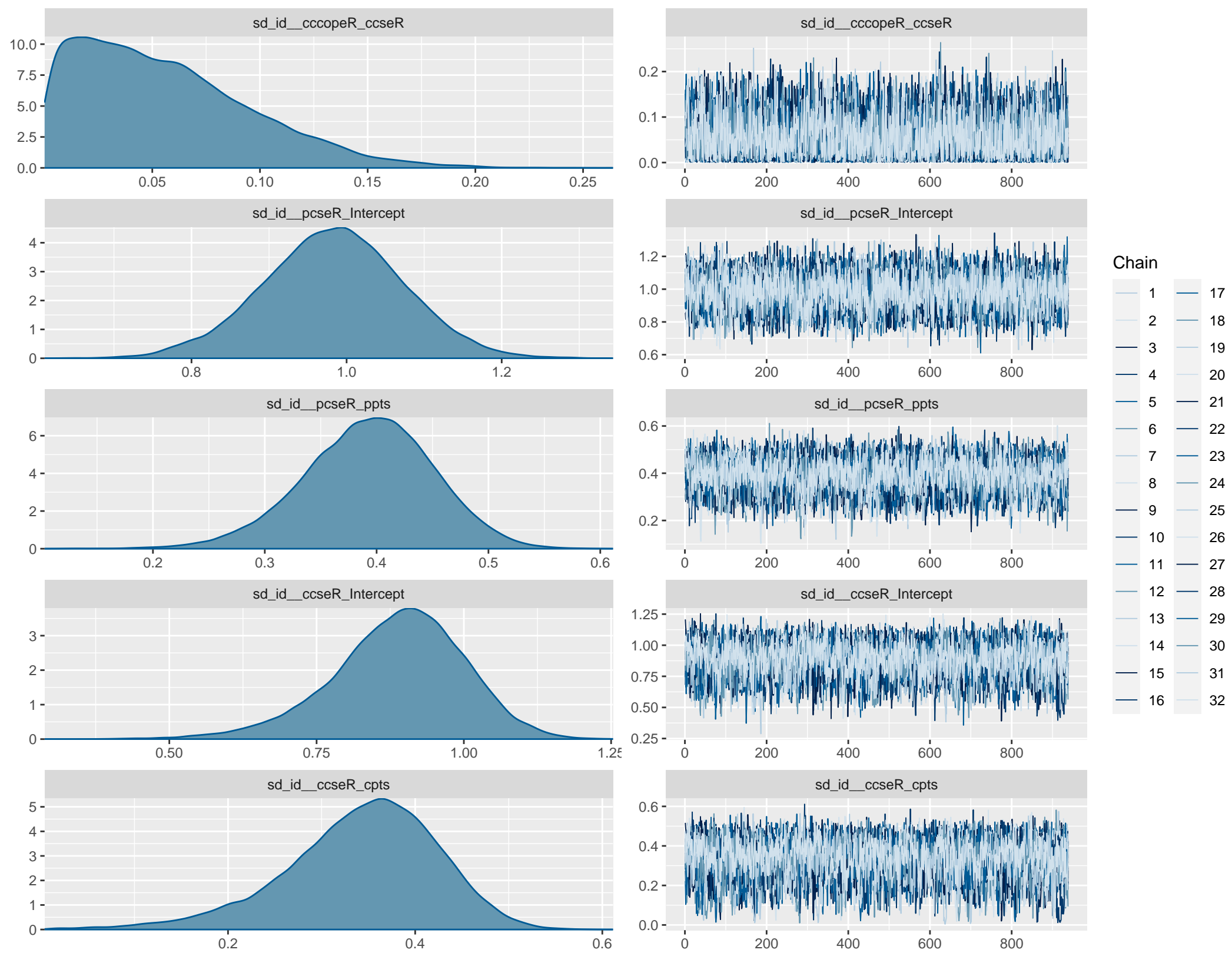

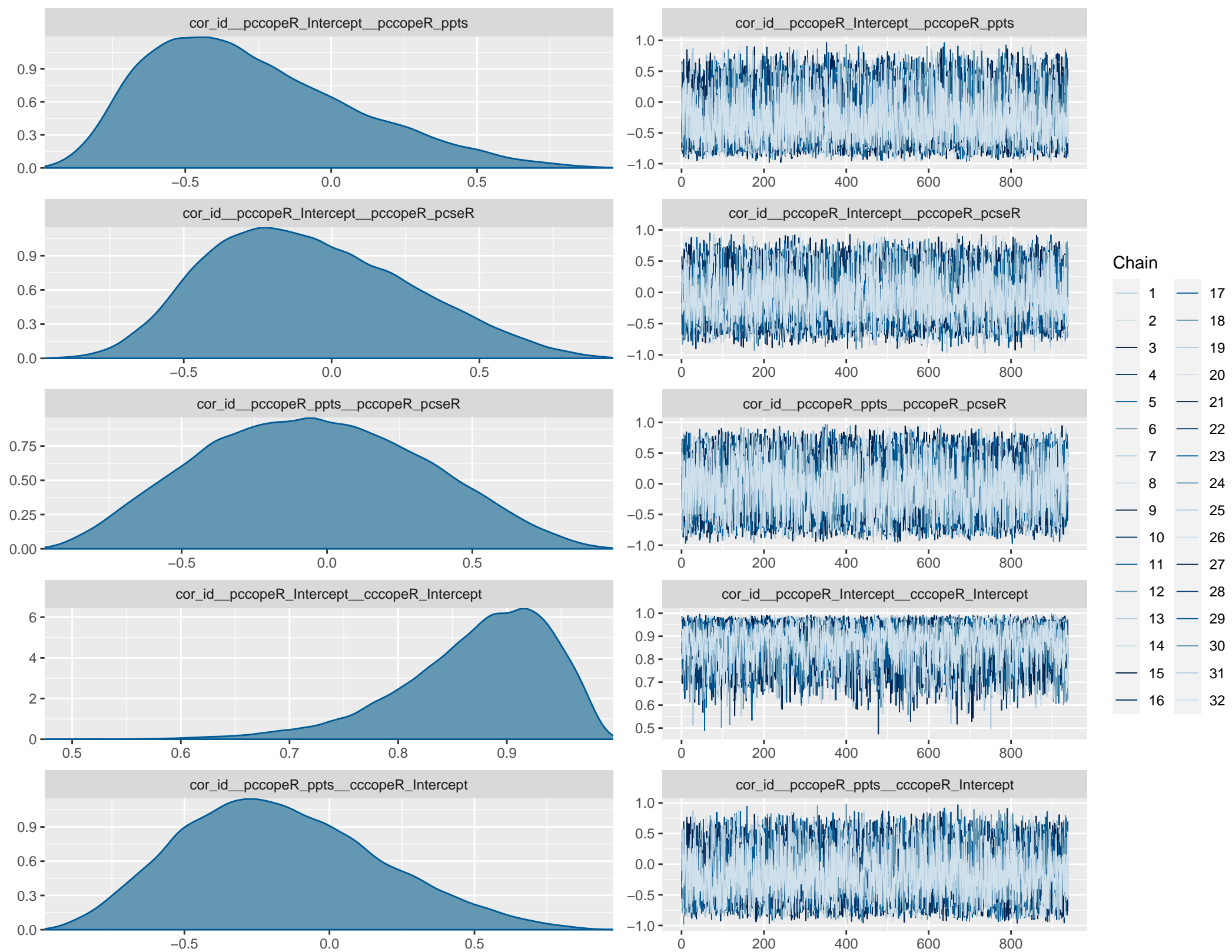

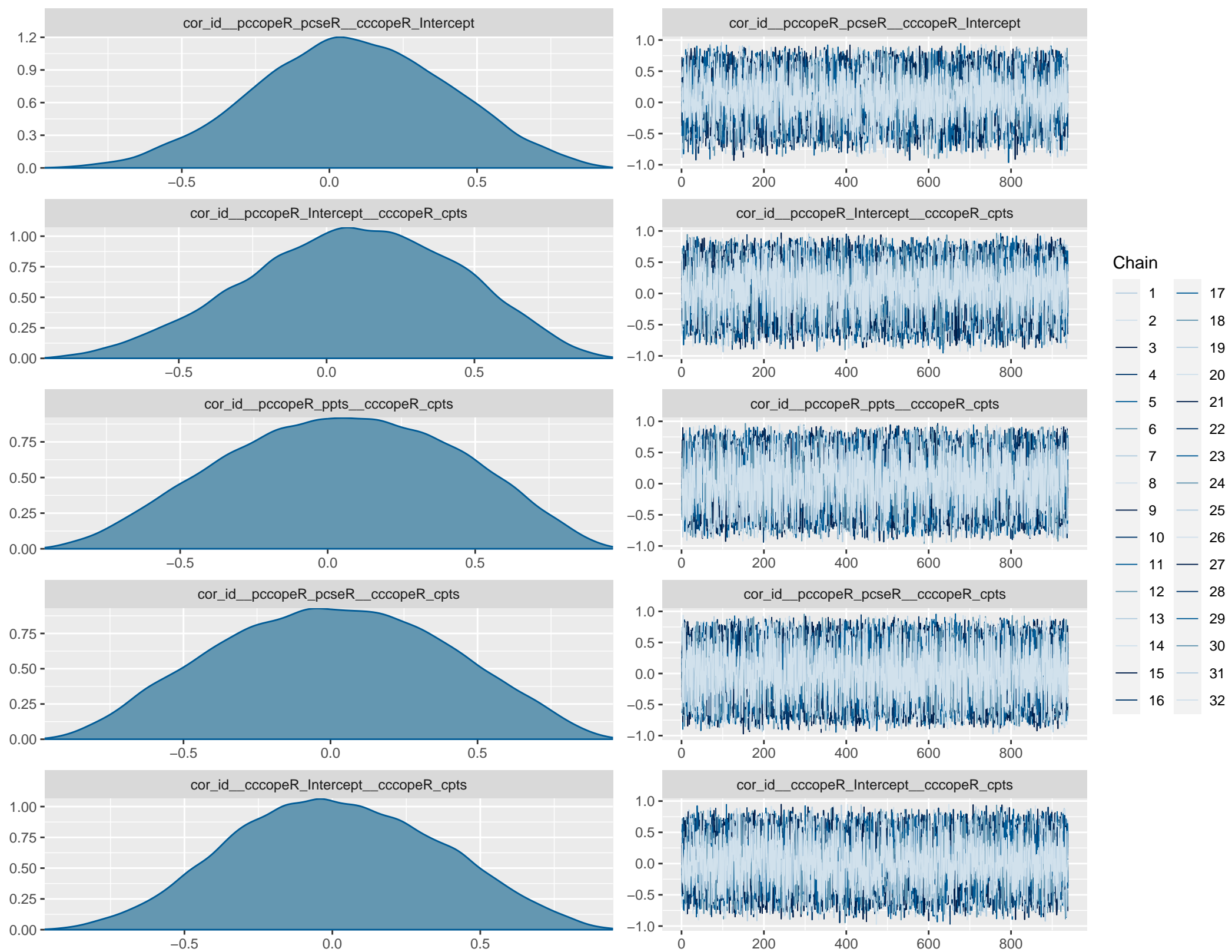

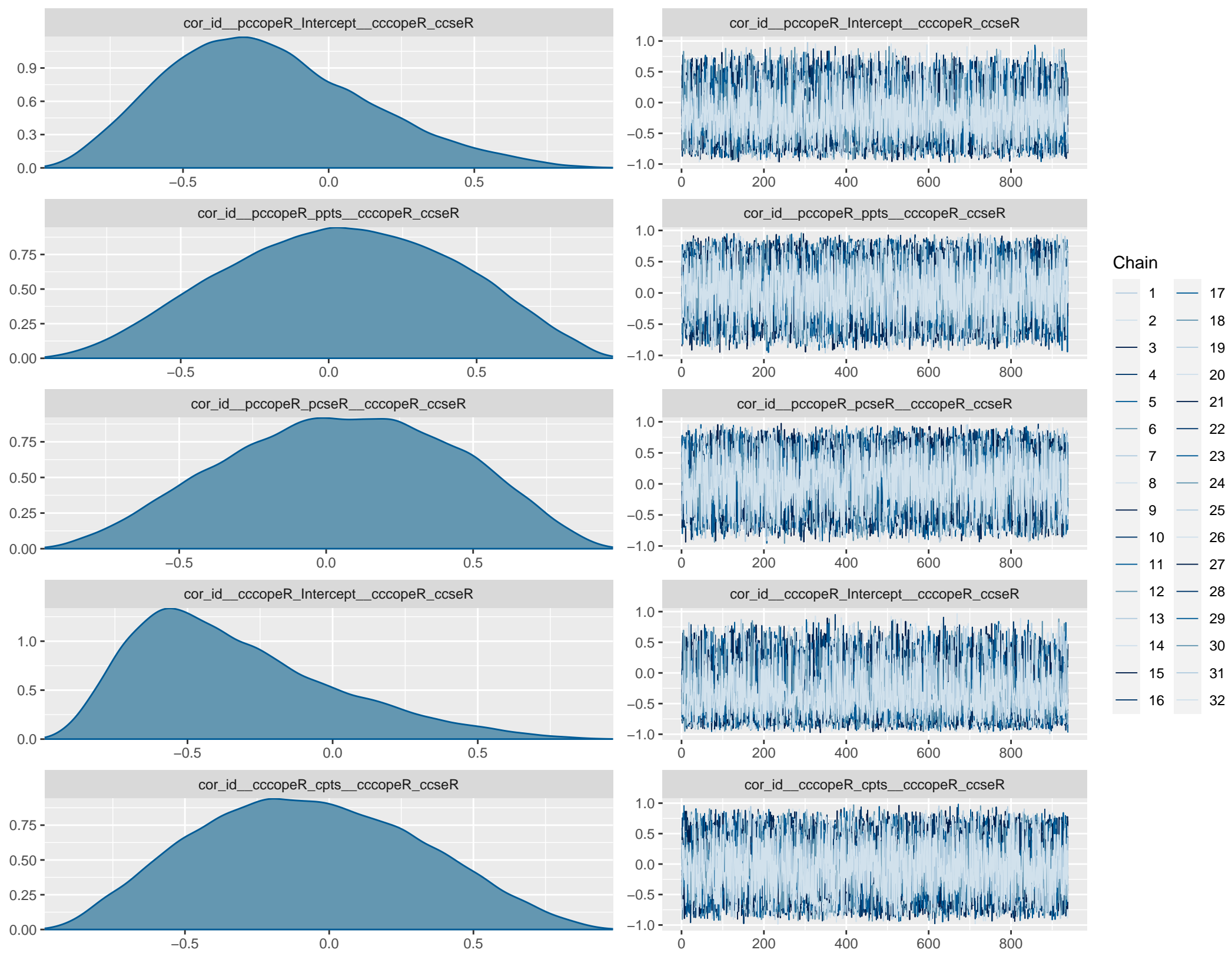

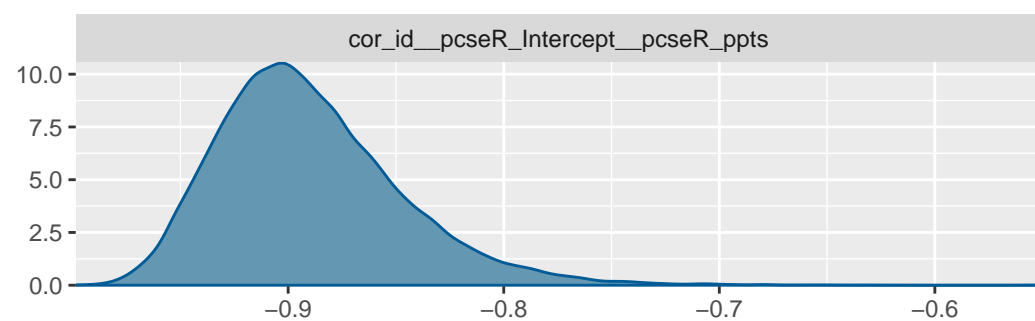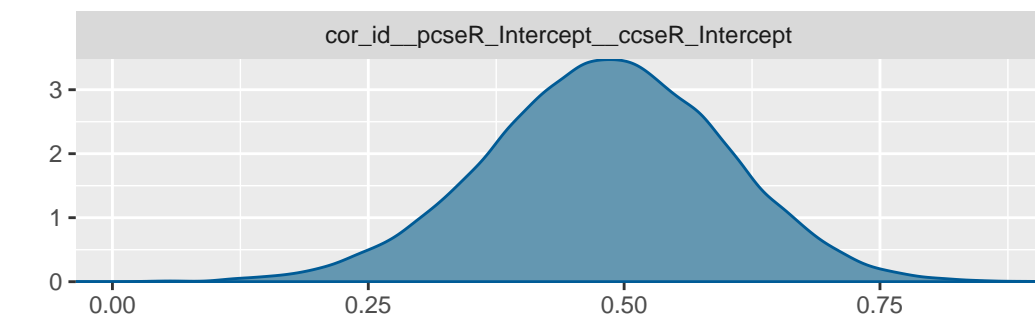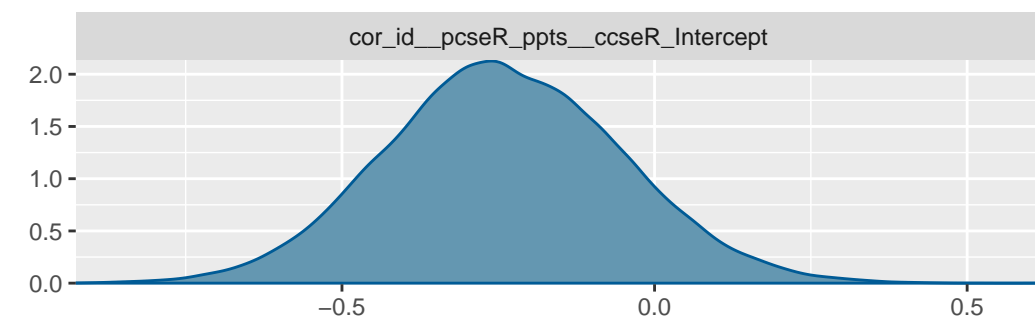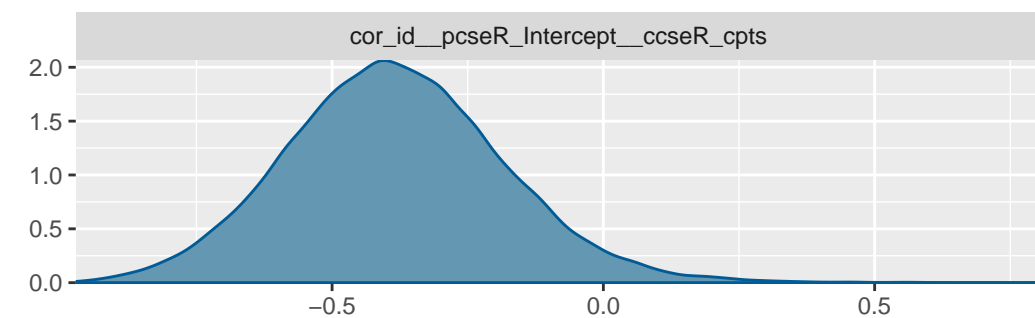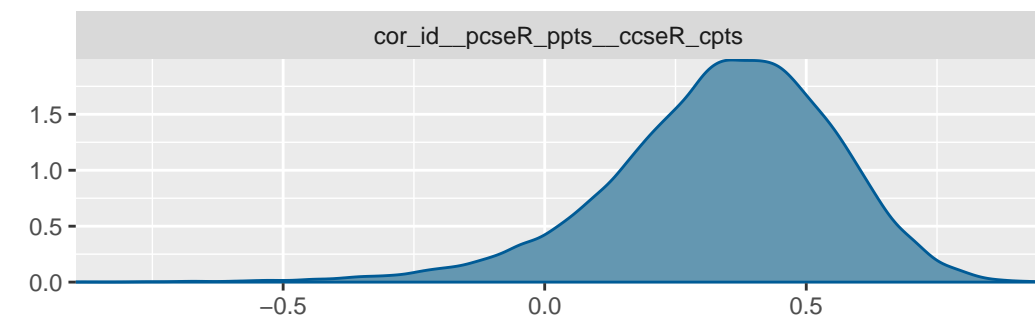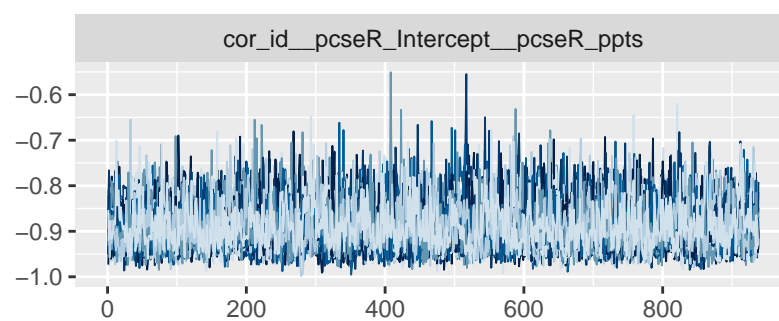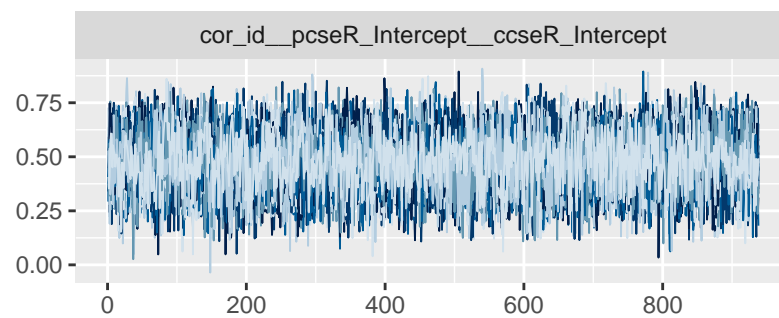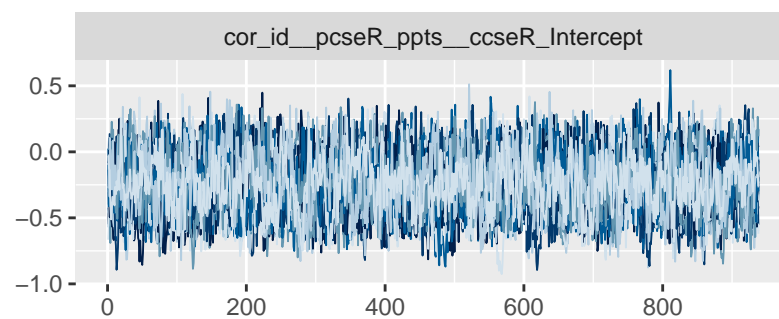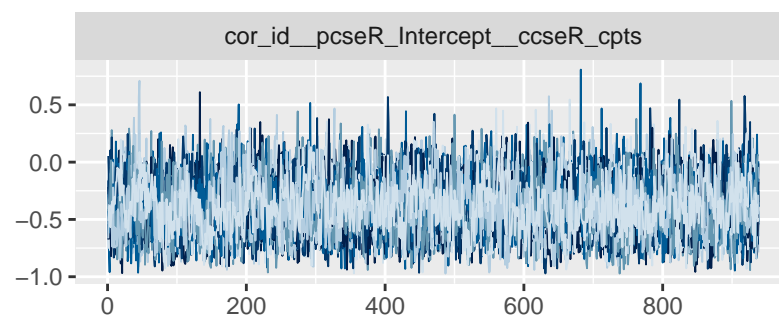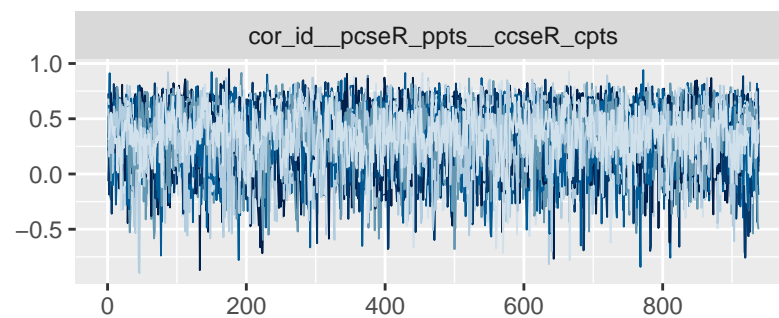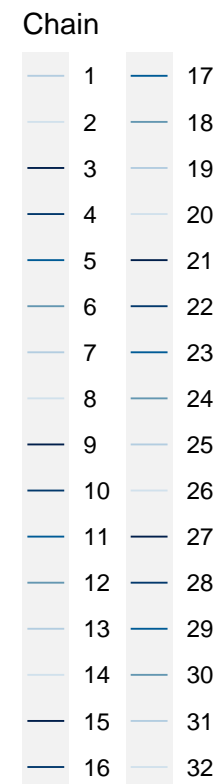

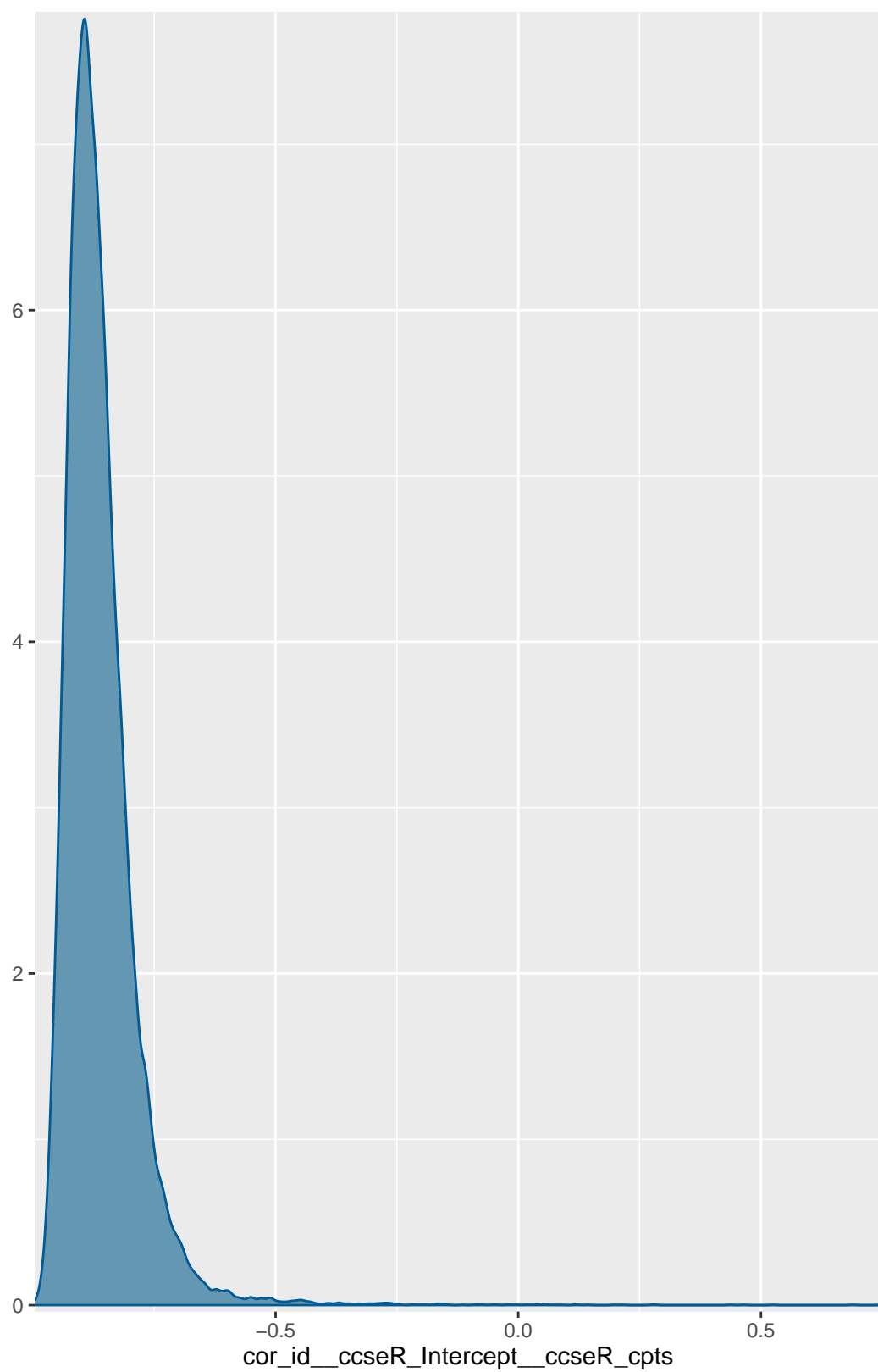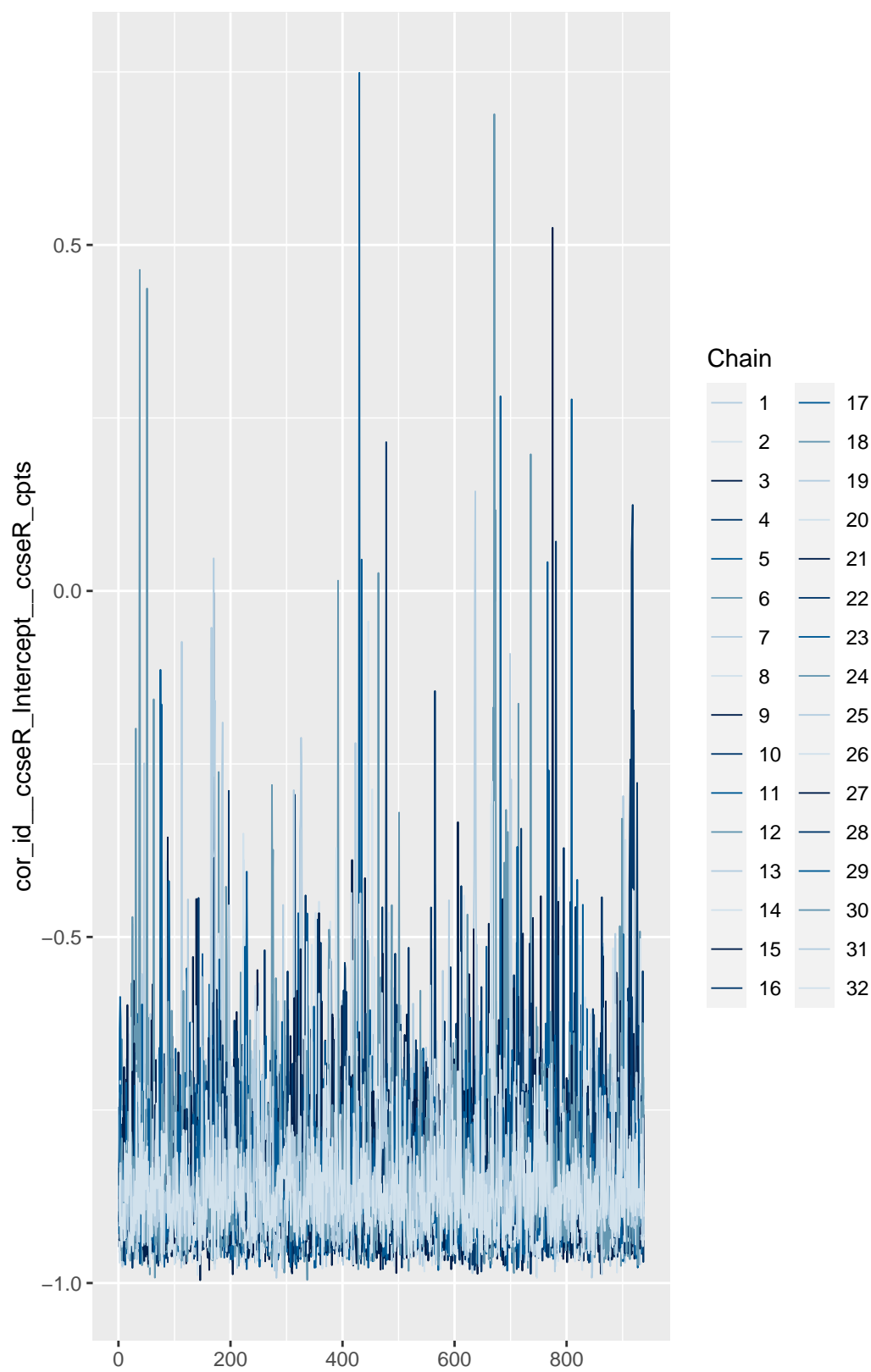

Supplement: 2 [file NIHMS1968016-supplement-2.pdf]
