# Supplementary material for: Coping self-efficacy mediates effects of posttraumatic distress on communal coping in parent-adolescence dyads after floods
Source: Dev Psychopathol. Author manuscript; Available in PMC 2025 Nov 1. (PMC11401968; doi:10.1017/S0954579424000567)
Supplement: 3 [file NIHMS1968016-supplement-3.pdf]

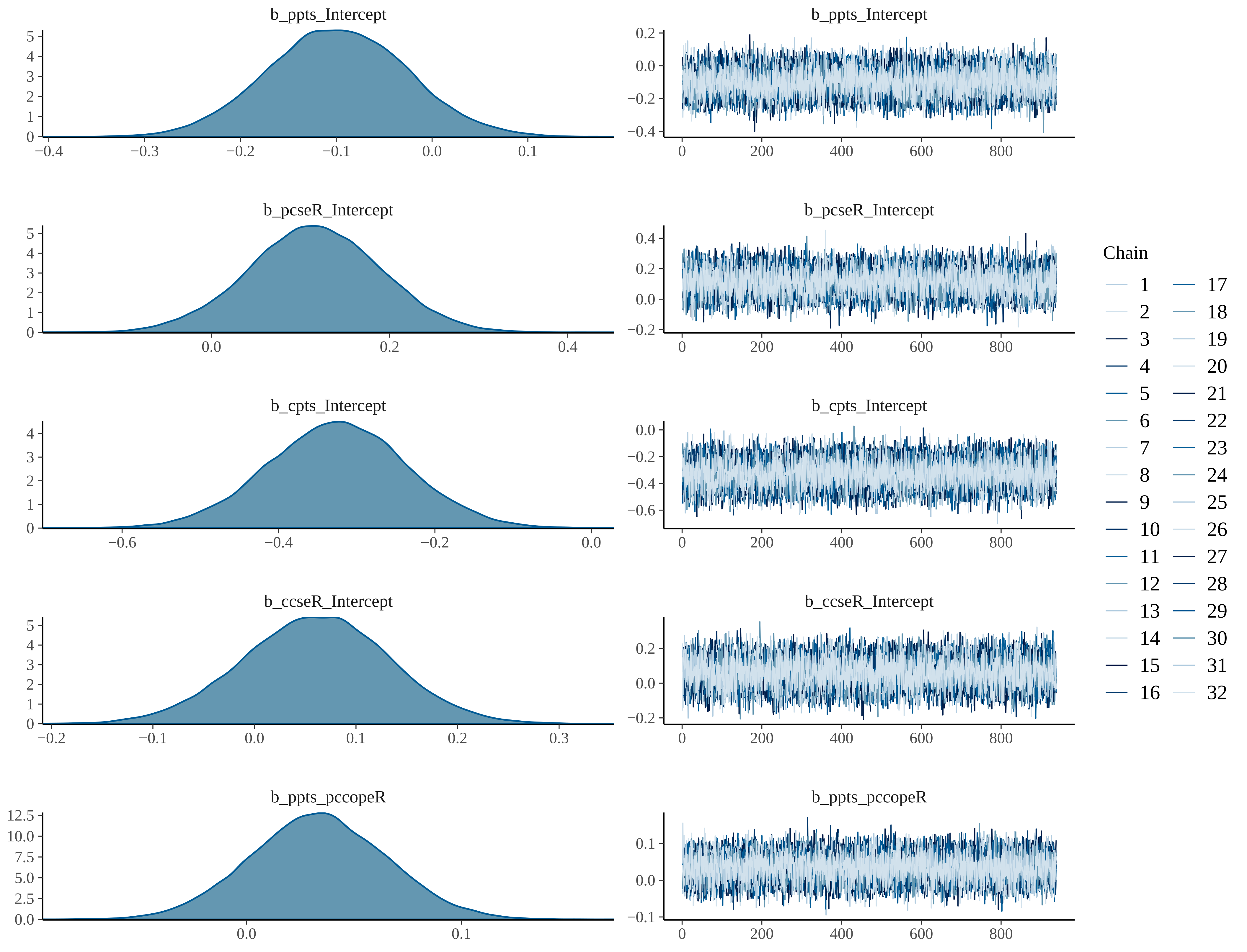
b_ppts_Intercept
b_pcseR_Intercept
b_cpts_Intercept
b_ccseR_Intercept
b_ppts_pccopeR
Chain
1 2 3 4 5 6 7 8 9 10 11 12 13 14 15 16 17 18 19 20 21 22 23 24 25 26 27 28 29 30 31 32

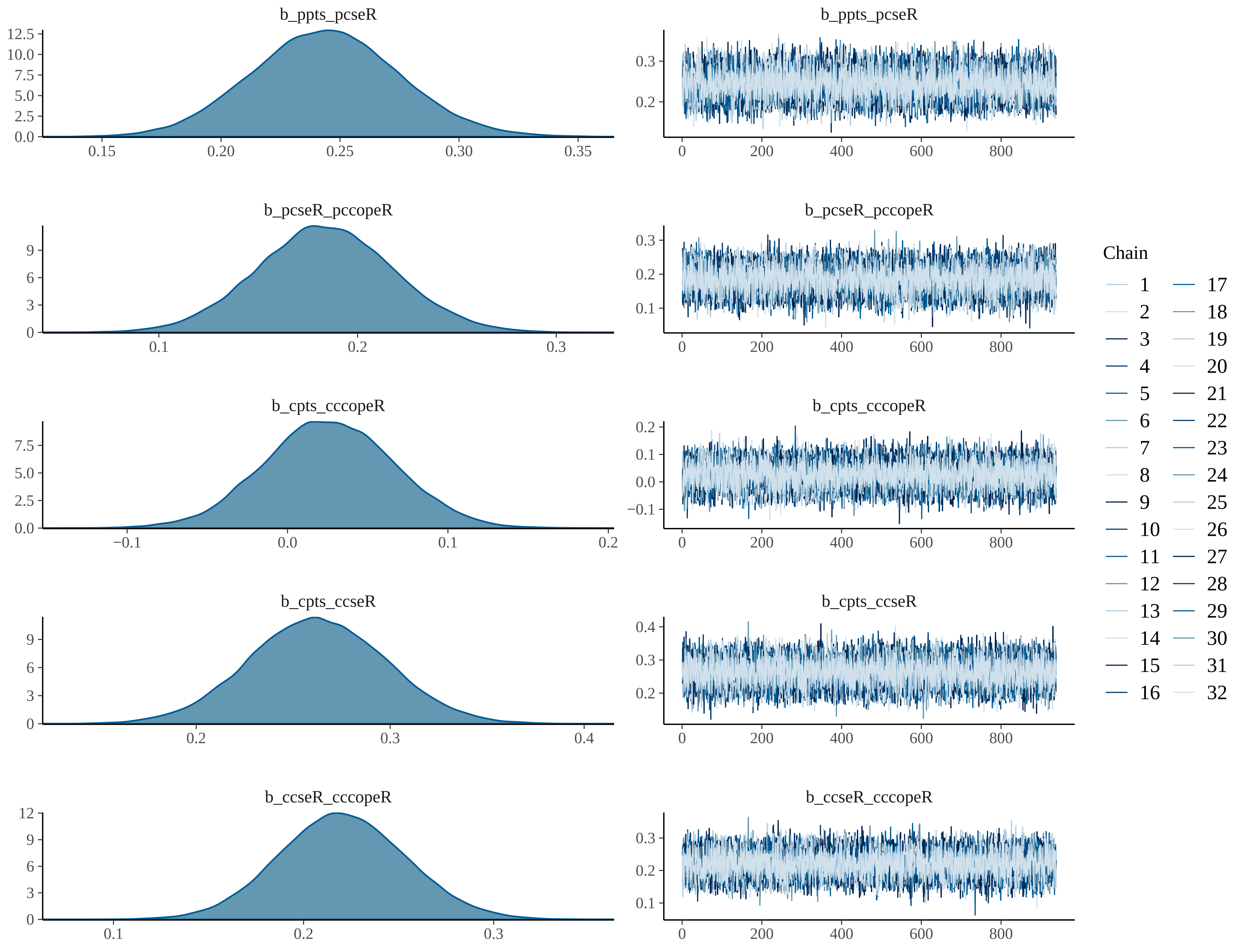
b_ppts_pcseR
b_pcseR_pccopeR
b_cpts_cccopeR
b_cpts_ccseR
b_ccseR_cccopeR
Chain
1 2 3 4 5 6 7 8 9 10 11 12 13 14 15 16 17 18 19 20 21 22 23 24 25 26 27 28 29 30 31 32

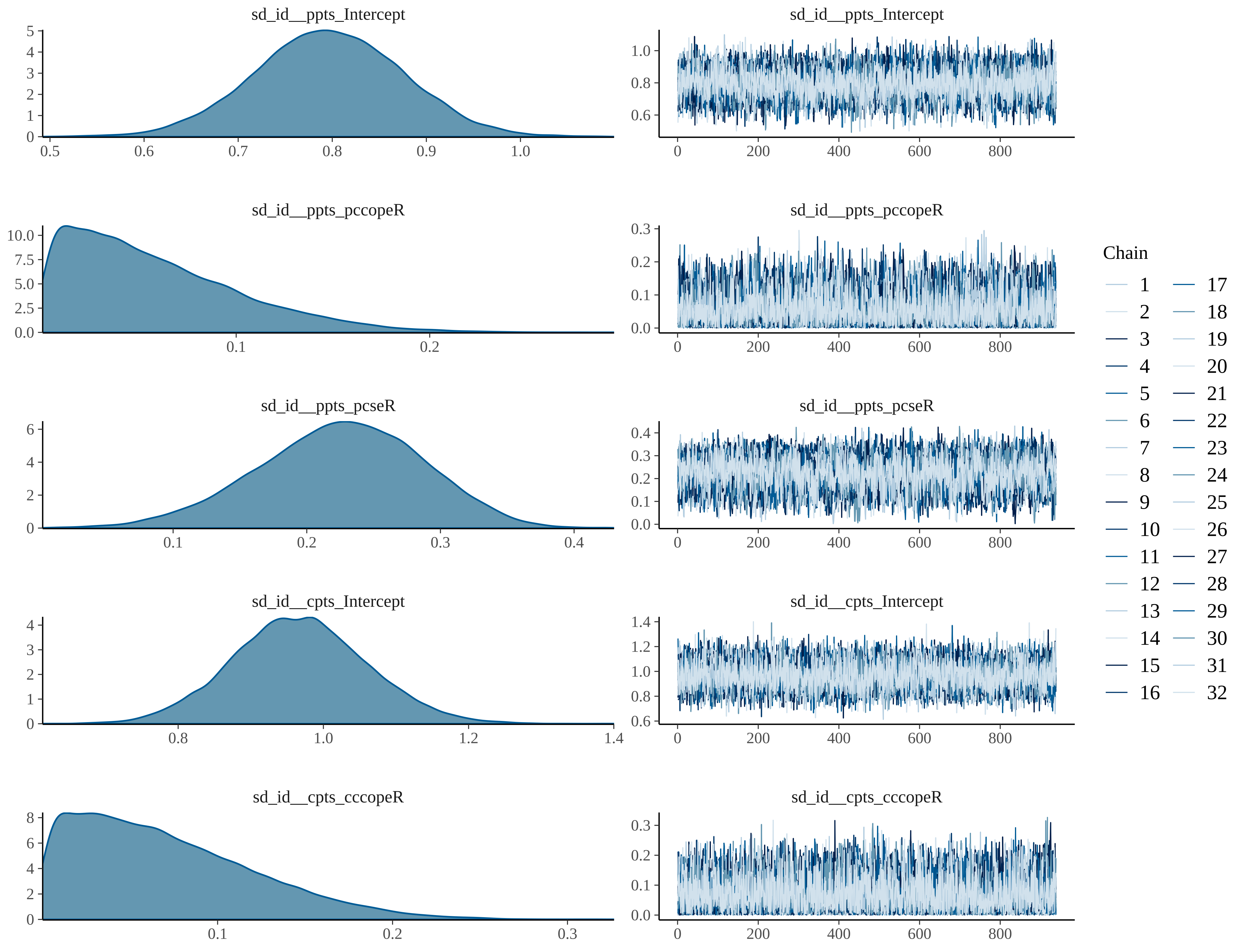
sd_id__ppts_Intercept
sd_id__ppts_pccopeR
sd_id__ppts_pcseR
sd_id__cpts_Intercept
sd_id__cpts_cccopeR
Chain
1 2 3 4 5 6 7 8 9 10 11 12 13 14 15 16 17 18 19 20 21 22 23 24 25 26 27 28 29 30 31 32

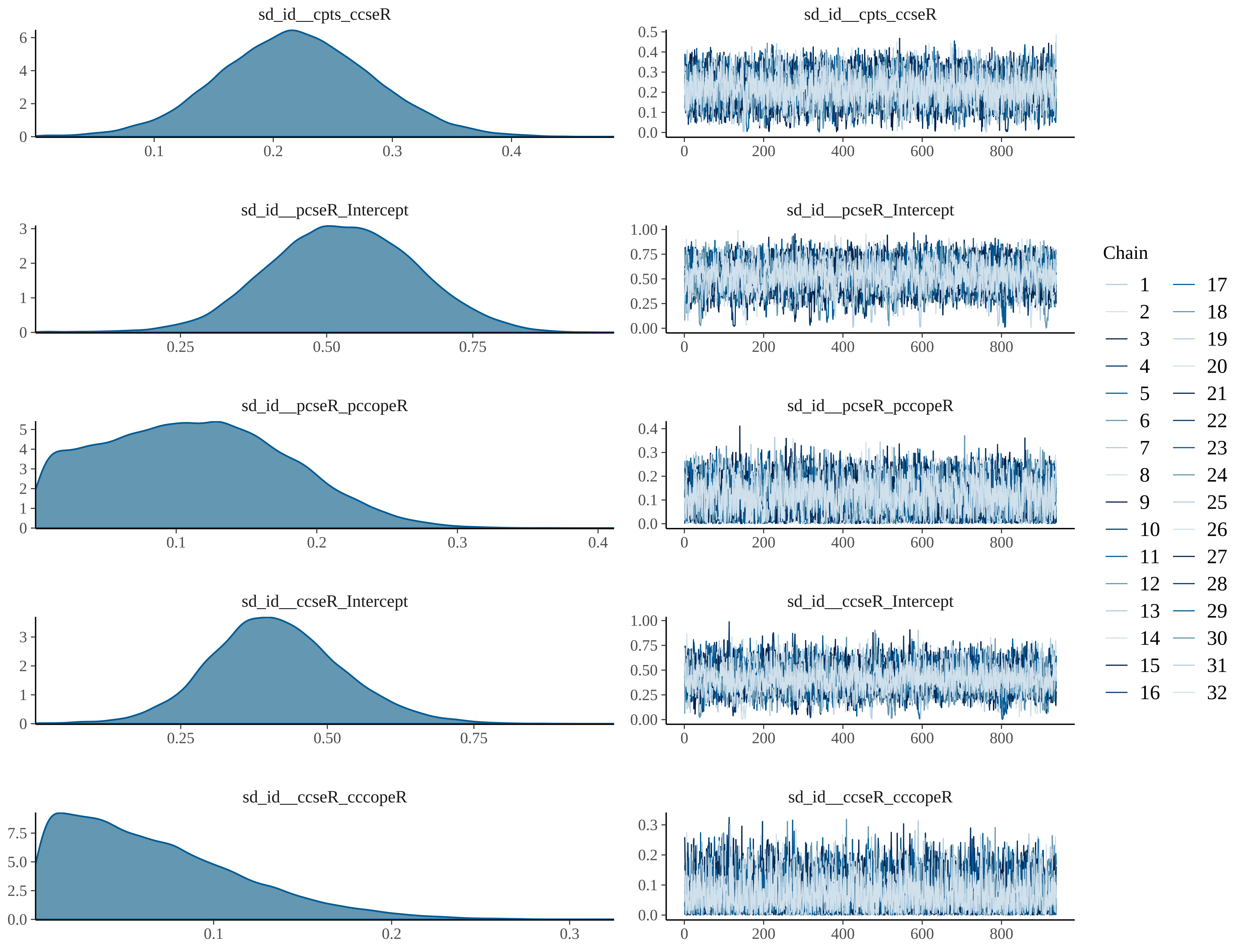
sd_id__cpts_ccseR
sd_id__pcseR_Intercept
sd_id__pcseR_pccopeR
sd_id__ccseR_Intercept
sd_id__ccseR_cccopeR
Chain
1 2 3 4 5 6 7 8 9 10 11 12 13 14 15 16 17 18 19 20 21 22 23 24 25 26 27 28 29 30 31 32

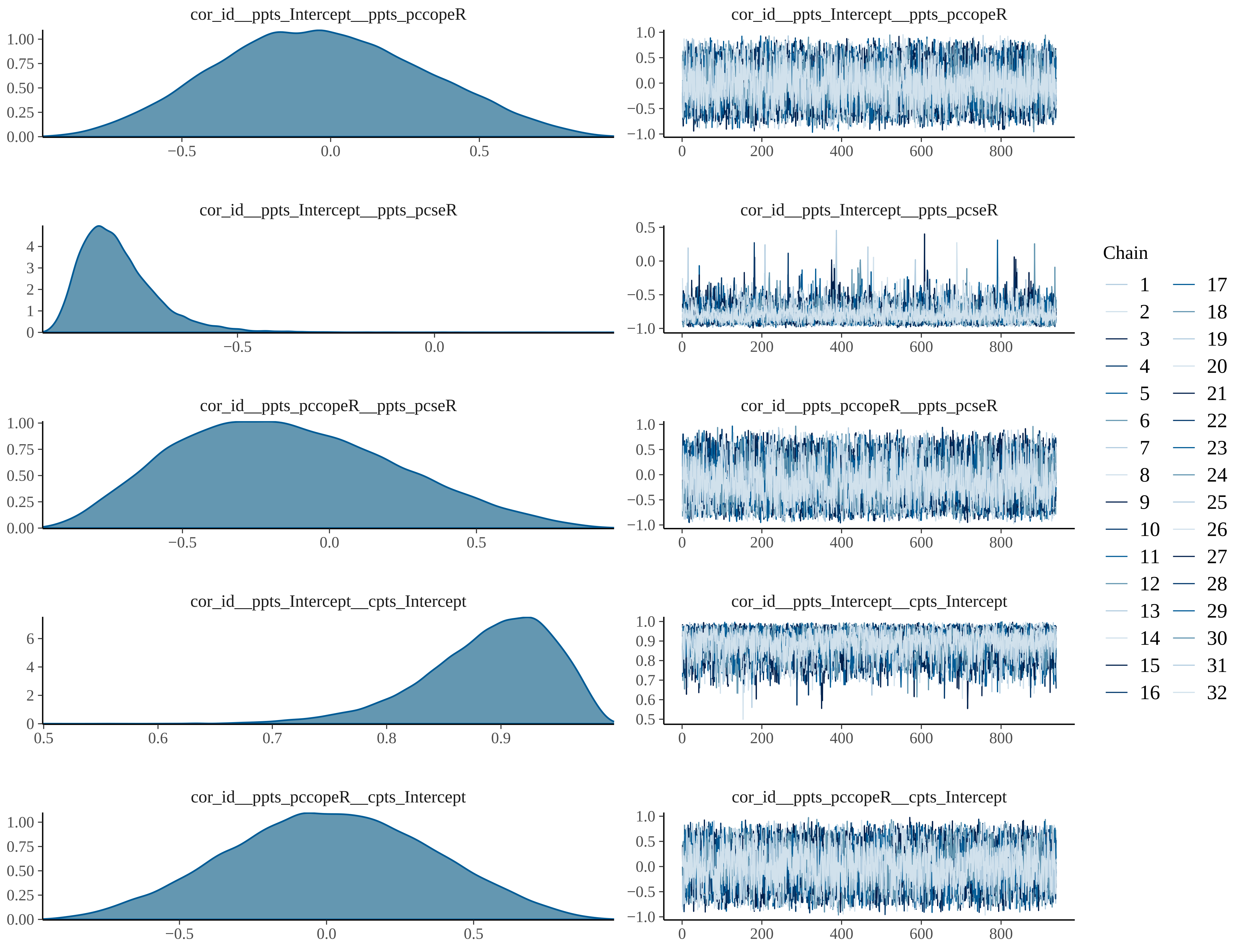

cor_id__ppts_Intercept__ppts_pccopeR
cor_id__ppts_Intercept__ppts_pcseR
cor_id__ppts_pccopeR__ppts_pcseR
cor_id__ppts_Intercept__cpts_Intercept
cor_id__ppts_pccopeR__cpts_Intercept
Chain
1 2 3 4 5 6 7 8 9 10 11 12 13 14 15 16 17 18 19 20 21 22 23 24 25 26 27 28 29 30 31 32

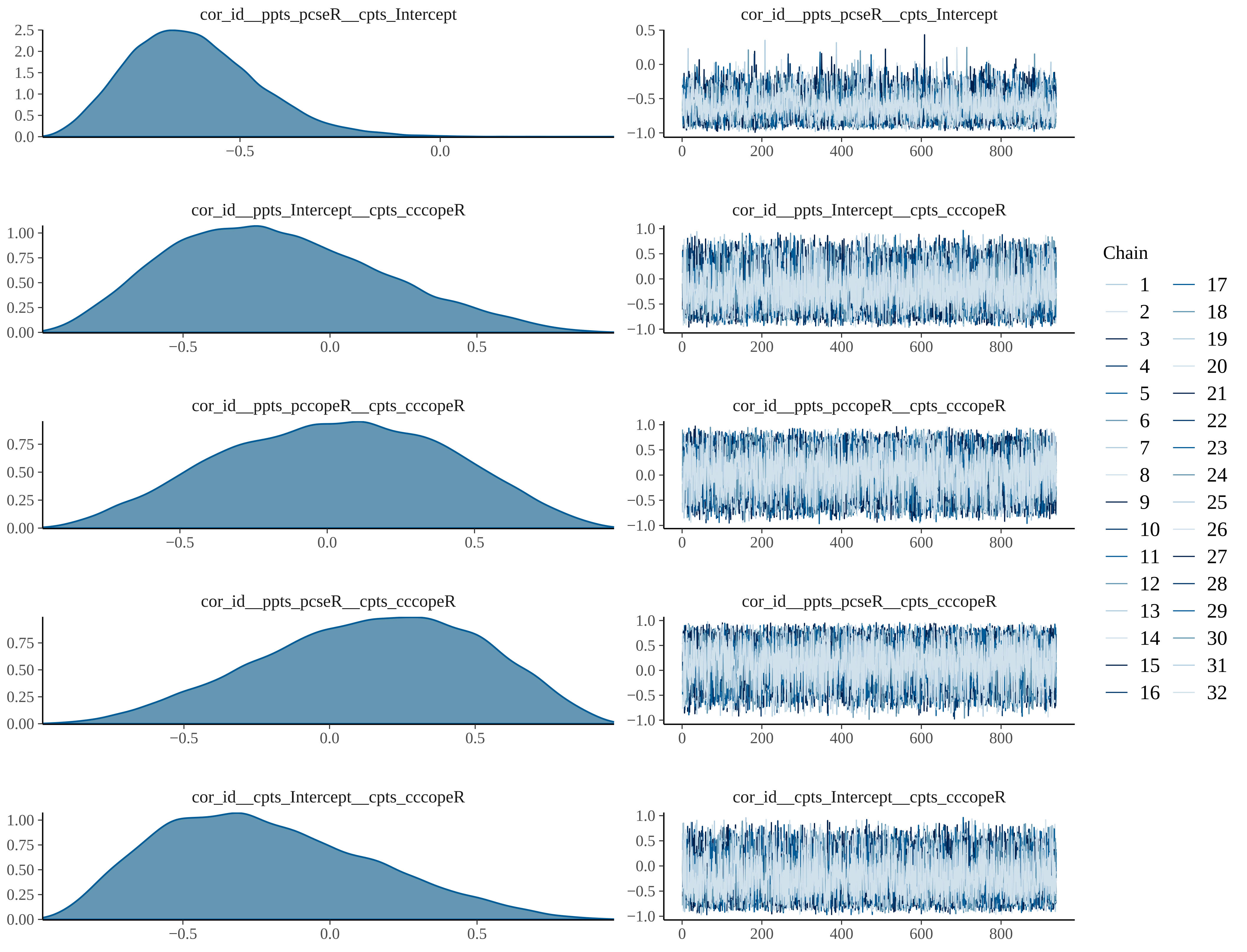

cor_id__ppts_pcseR__cpts_Intercept
cor_id__ppts_Intercept__cpts_cccopeR
cor_id__ppts_pccopeR__cpts_cccopeR
cor_id__ppts_pcseR__cpts_cccopeR
cor_id__cpts_Intercept__cpts_cccopeR
Chain
1
2
3
4
5
6
7
8
9
10
11
12
13
14
15
16
17
18
19
20
21
22
23
24
25
26
27
28
29
30
31
32

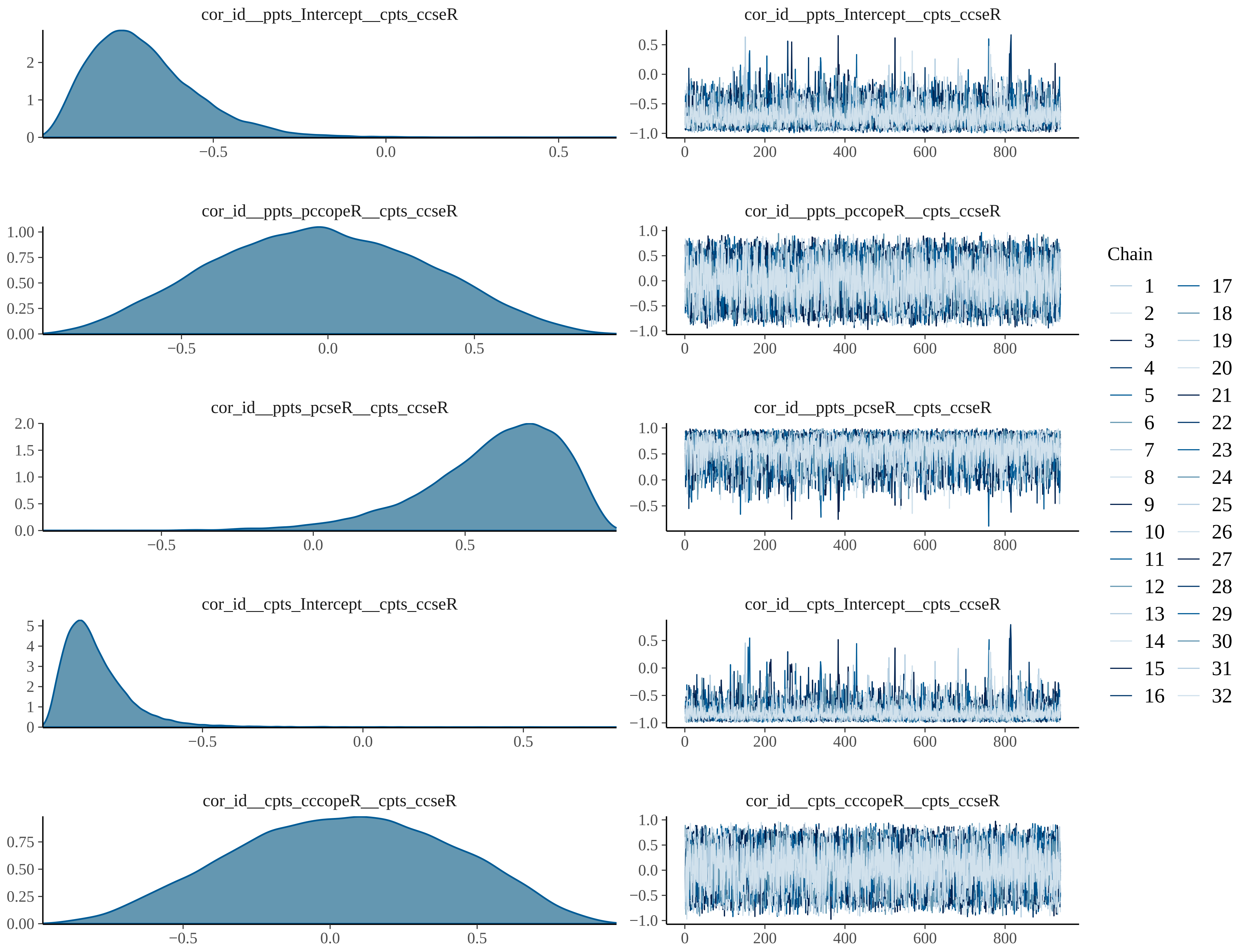
cor_id__ppts_Intercept__cpts_ccseR
cor_id__ppts_pccopeR__cpts_ccseR
cor_id__ppts_pcseR__cpts_ccseR
cor_id__cpts_Intercept__cpts_ccseR
cor_id__cpts_cccopeR__cpts_ccseR
Chain
1
2
3
4
5
6
7
8
9
10
11
12
13
14
15
16
17
18
19
20
21
22
23
24
25
26
27
28
29
30
31
32

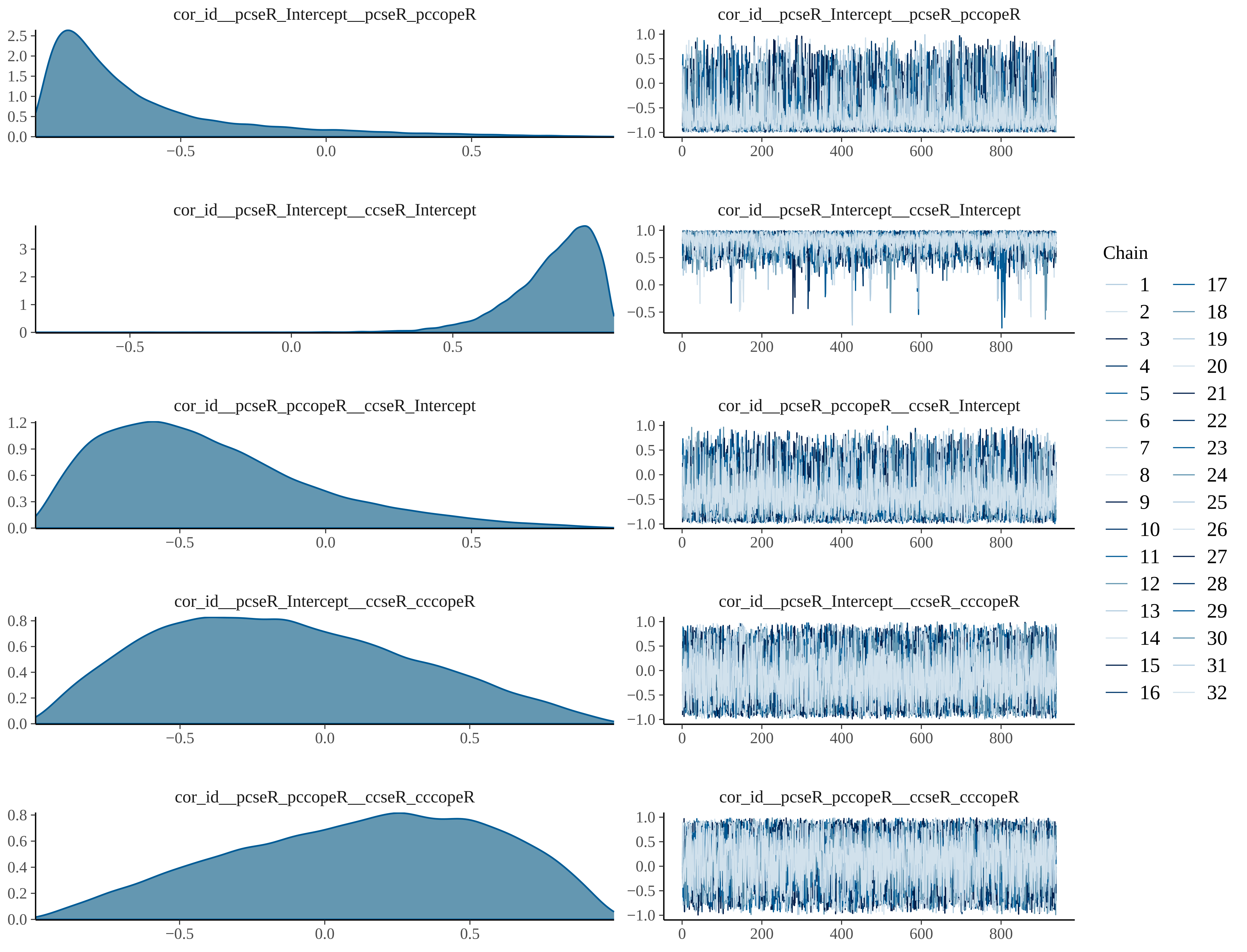
cor_id__pcseR_Intercept__pcseR_pccopeR
cor_id__pcseR_Intercept__ccseR_Intercept
cor_id__pcseR_pccopeR__ccseR_Intercept
cor_id__pcseR_Intercept__ccseR_cccopeR
cor_id__pcseR_pccopeR__ccseR_cccopeR
Chain
1
2
3
4
5
6
7
8
9
10
11
12
13
14
15
16
17
18
19
20
21
22
23
24
25
26
27
28
29
30
31
32

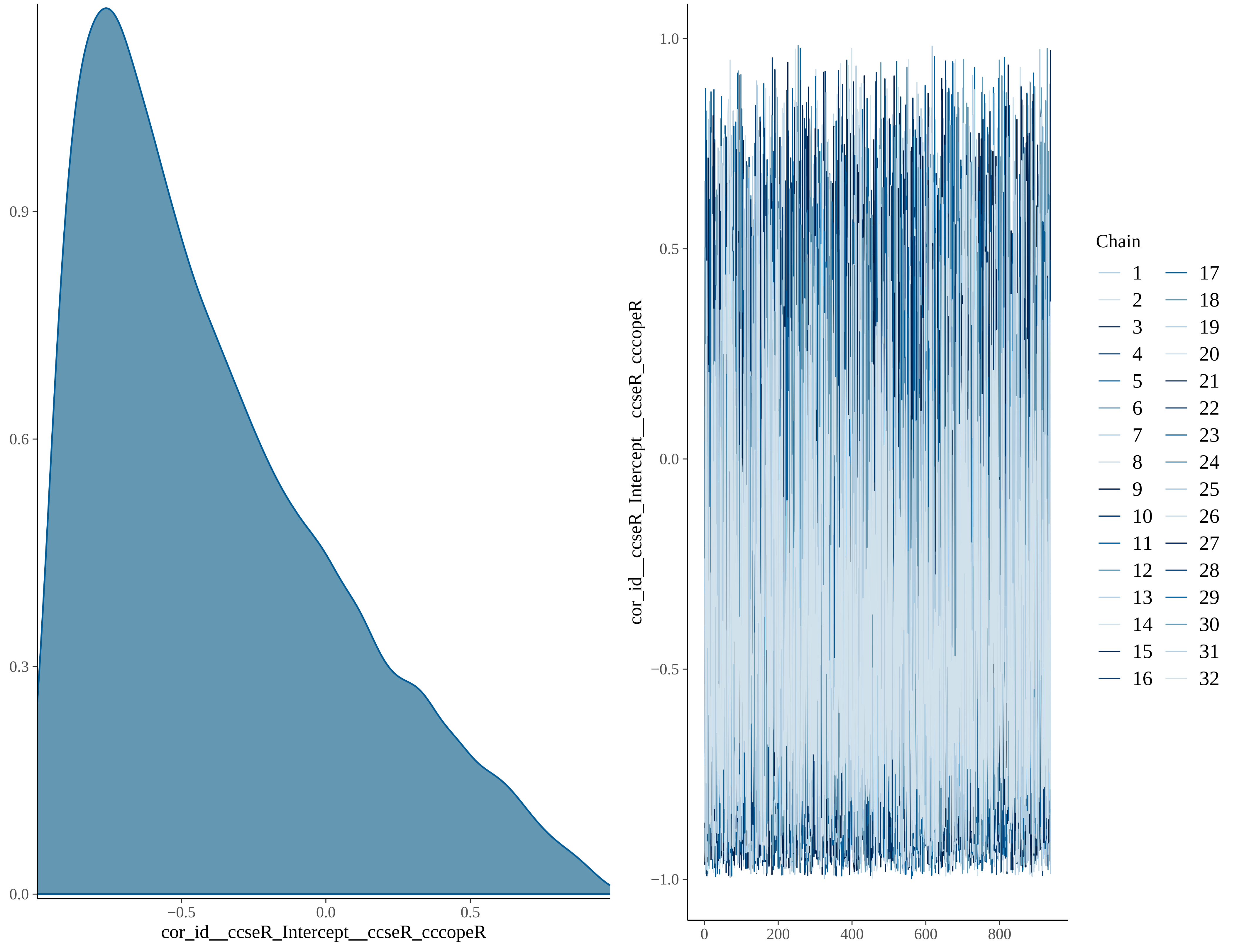

0.9
0.6
0.3
0.0
−0.5
0.0
0.5
cor_id__ccseR_Intercept__ccseR_ccccopeR
1.0
0.5
0.0
−0.5
−1.0
cor_id__ccseR_Intercept__ccseR_ccccopeR
0
200
400
600
800
Chain
1
2
3
4
5
6
7
8
9
10
11
12
13
14
15
16
17
18
19
20
21
22
23
24
25
26
27
28
29
30
31
32
