# Supplementary material for: Coping self-efficacy mediates effects of posttraumatic distress on communal coping in parent-adolescence dyads after floods
Source: Dev Psychopathol. Author manuscript; Available in PMC 2025 Nov 1. (PMC11401968; doi:10.1017/S0954579424000567)
Supplement: 4 [file NIHMS1968016-supplement-4.pdf]

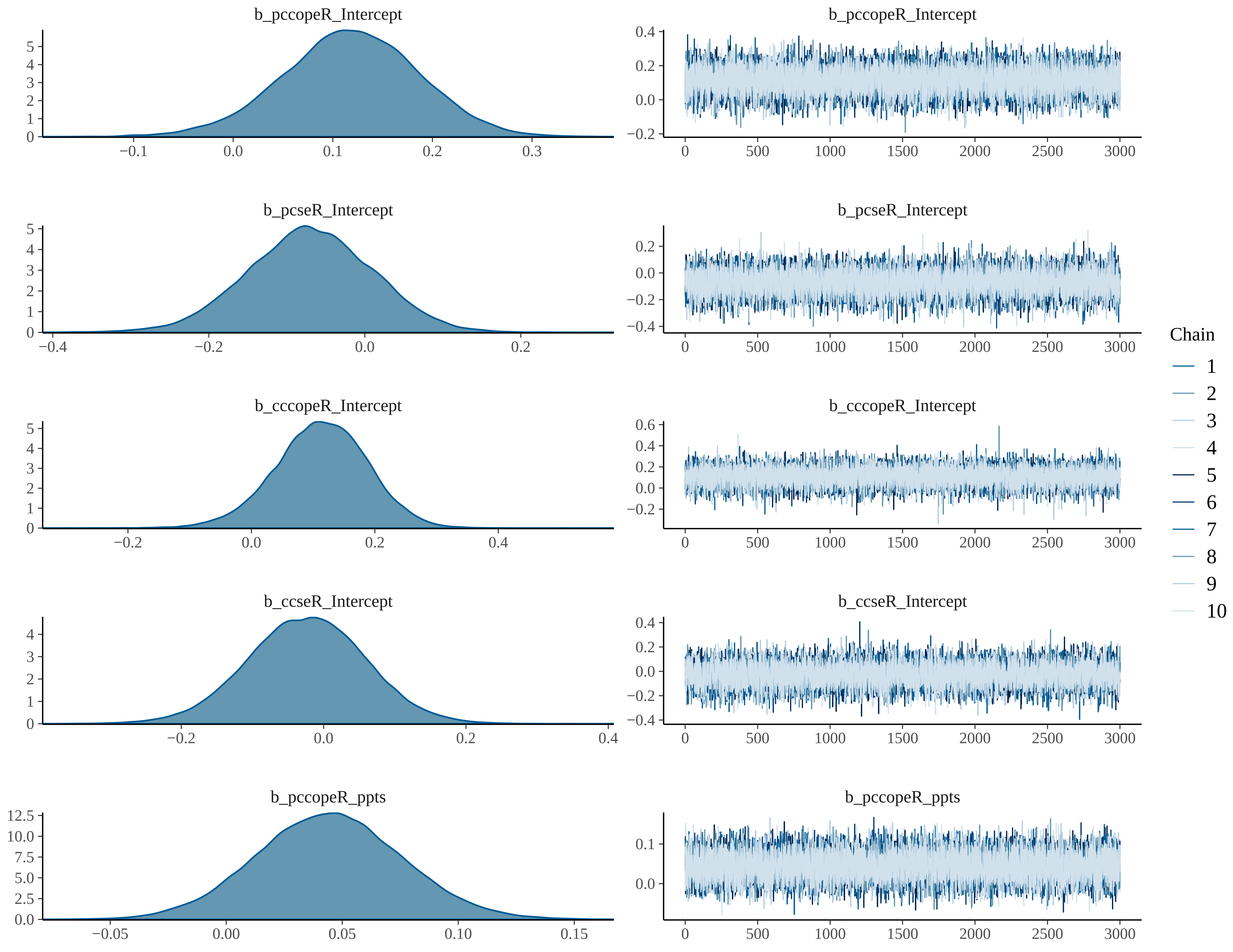

b_pccopeR_Intercept
b_pcseR_Intercept
b_cccopeR_Intercept
b_ccseR_Intercept
b_pccopeR_ppts
Chain
1
2
3
4
5
6
7
8
9
10

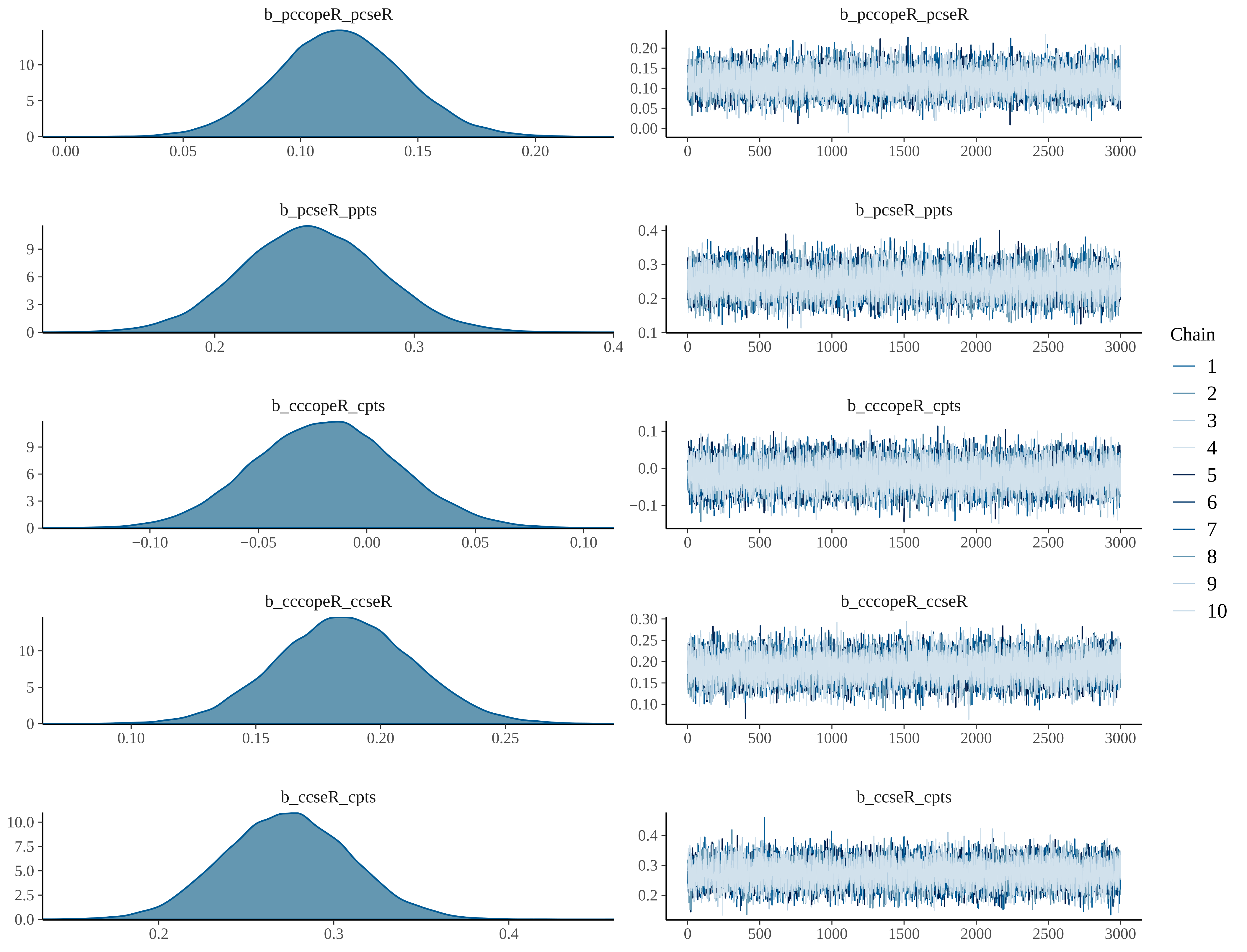

b_pccopeR_pcseR
b_pcseR_ppts
b_cccopeR_cpts
b_cccopeR_ccseR
b_ccseR_cpts
Chain
1
2
3
4
5
6
7
8
9
10

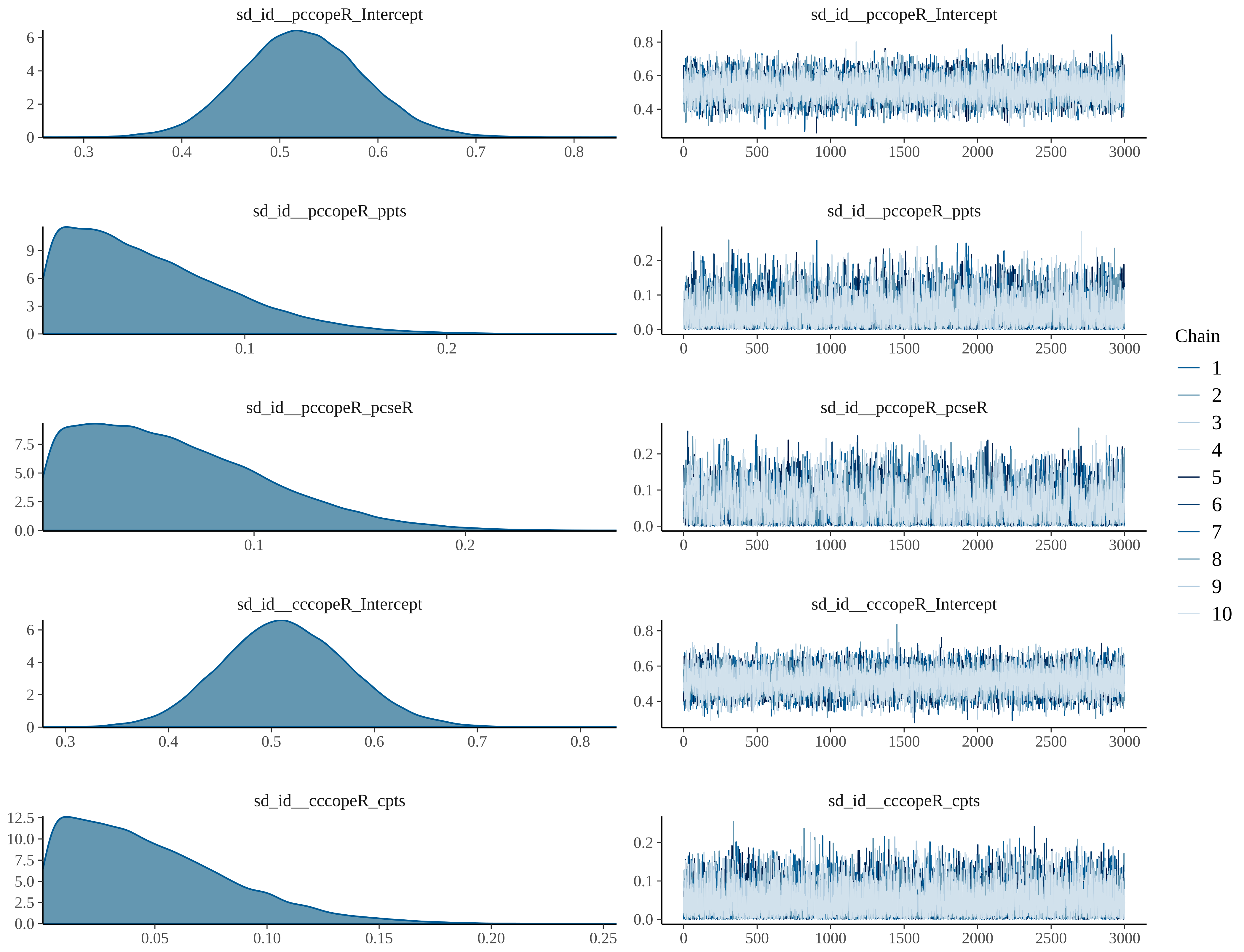
sd_id__pccopeR_Intercept
sd_id__pccopeR_ppts
sd_id__pccopeR_pcseR
sd_id__cccopeR_Intercept
sd_id__cccopeR_cpts
Chain
1
2
3
4
5
6
7
8
9
10

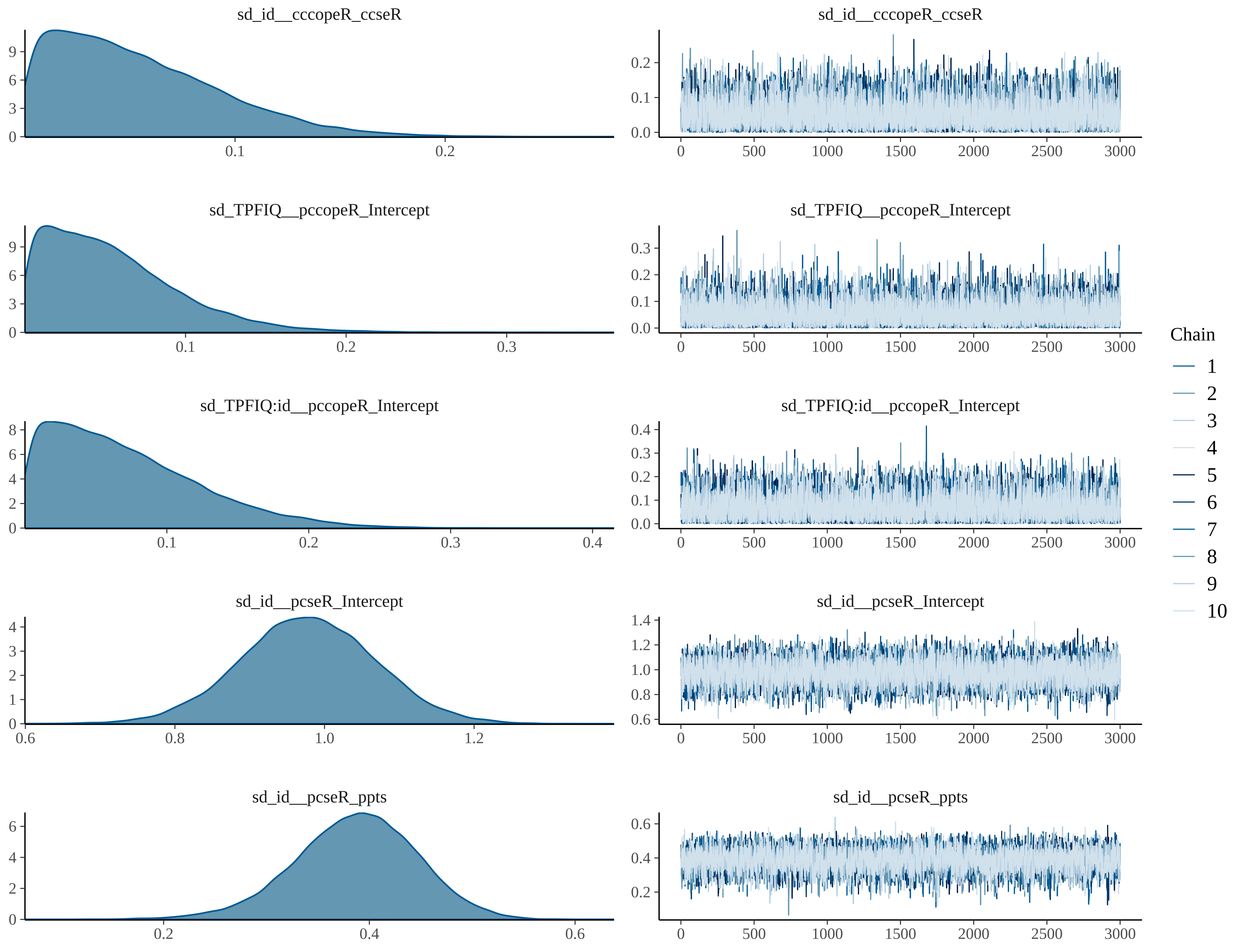

sd_id__cccopeR_ccseR
sd_TPFIQ__pccopeR_Intercept
sd_TPFIQ:id__pccopeR_Intercept
sd_id__pcseR_Intercept
sd_id__pcseR_ppts
Chain
1
2
3
4
5
6
7
8
9
10

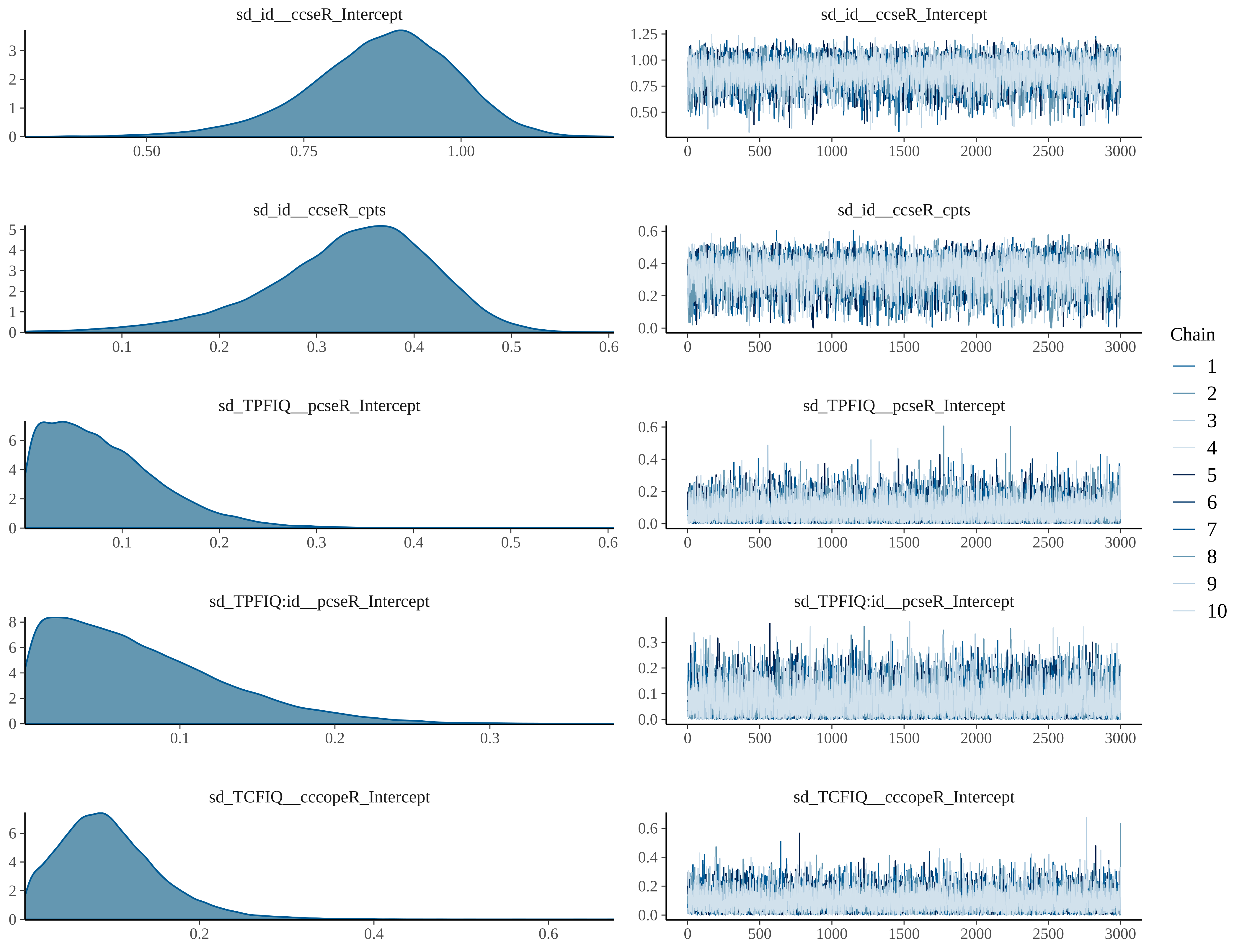

sd_id__ccseR_Intercept
sd_id__ccseR_cpts
sd_TPFIQ__pcseR_Intercept
sd_TPFIQ:id__pcseR_Intercept
sd_TCFIQ__cccopeR_Intercept
Chain
1
2
3
4
5
6
7
8
9
10

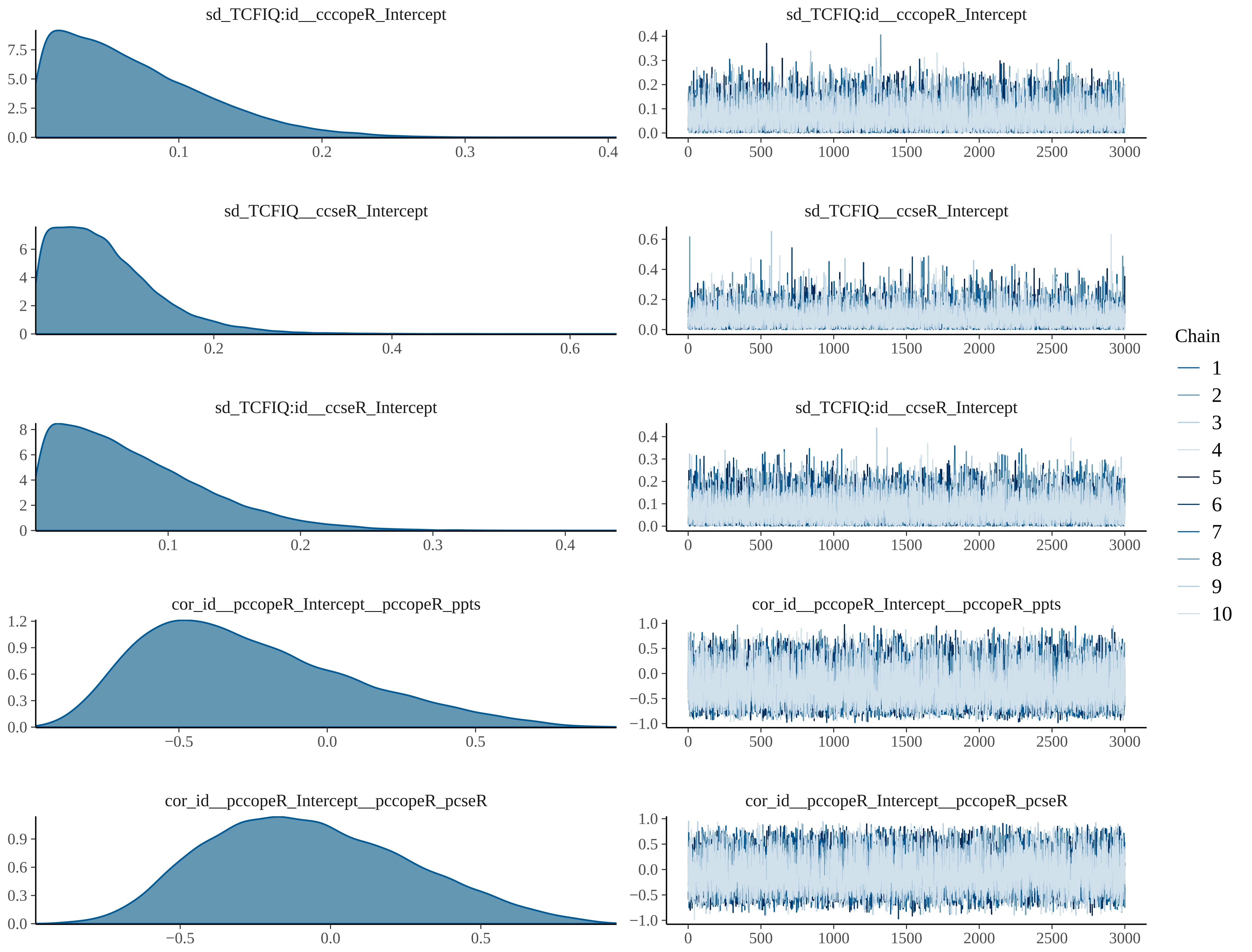
sd_TCFIQ:id__cccopeR_Intercept
sd_TCFIQ__ccseR_Intercept
sd_TCFIQ:id__ccseR_Intercept
cor_id__pccopeR_Intercept__pccopeR_ppts
cor_id__pccopeR_Intercept__pccopeR_pcseR
Chain
1
2
3
4
5
6
7
8
9
10

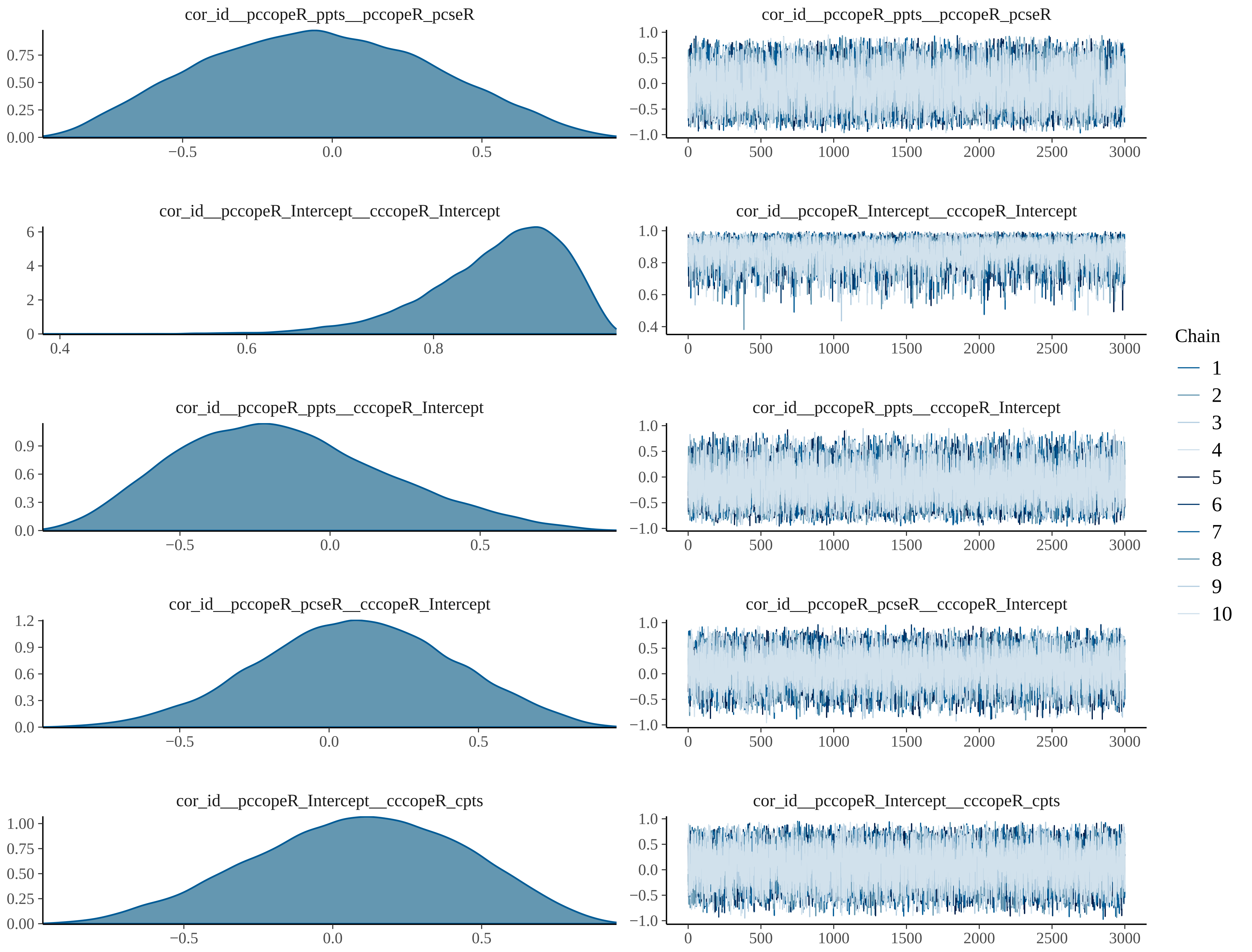

cor_id__pccopeR_ppts__pccopeR_pcseR
cor_id__pccopeR_Intercept__cccopeR_Intercept
cor_id__pccopeR_ppts__cccopeR_Intercept
cor_id__pccopeR_pcseR__cccopeR_Intercept
cor_id__pccopeR_Intercept__cccopeR_cpts
Chain
1
2
3
4
5
6
7
8
9
10

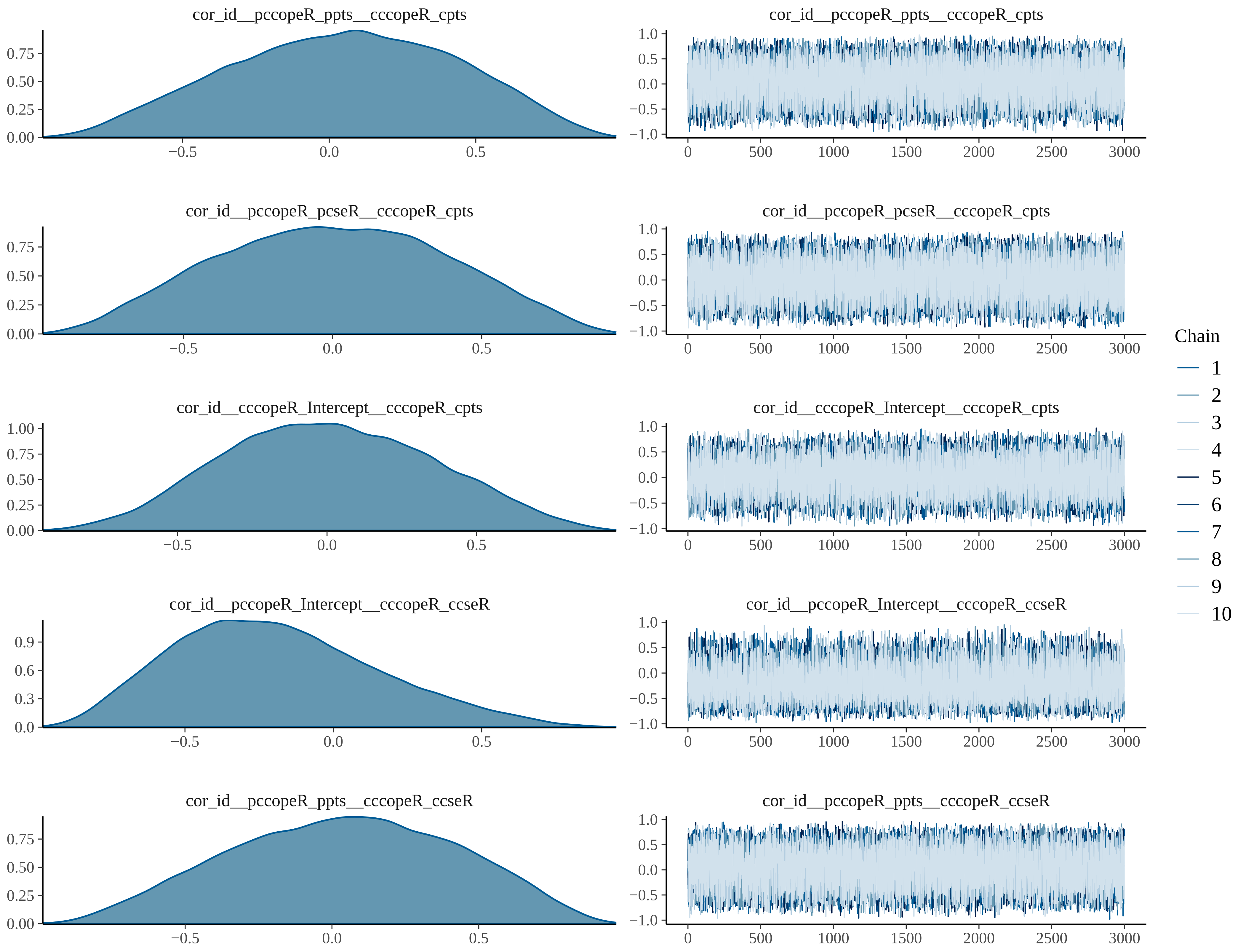
cor_id__pccopeR_ppts__cccopeR_cpts
cor_id__pccopeR_pcseR__cccopeR_cpts
cor_id__cccopeR_Intercept__cccopeR_cpts
cor_id__pccopeR_Intercept__cccopeR_ccseR
cor_id__pccopeR_ppts__cccopeR_ccseR
0.00
0.25
0.50
0.75
1.00
0.0
0.3
0.6
0.9
−0.5
0.0
0.5
1.0
−1.0
0
500
1000
1500
2000
2500
3000
Chain
1
2
3
4
5
6
7
8
9
10

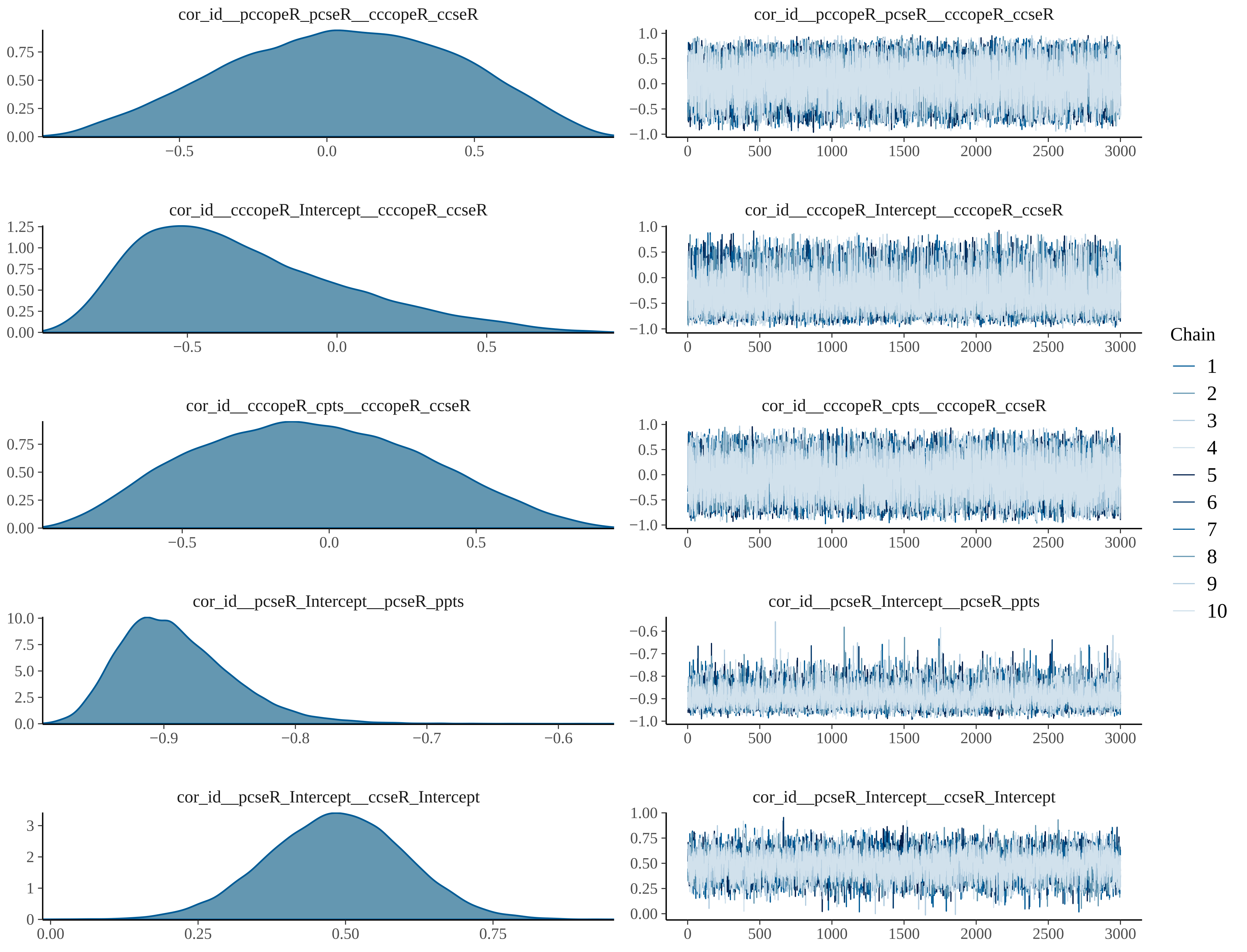

cor_id__pccopeR_pcseR__cccopeR_ccseR
cor_id__cccopeR_Intercept__cccopeR_ccseR
cor_id__cccopeR_cpts__cccopeR_ccseR
cor_id__pcseR_Intercept__pcseR_ppts
cor_id__pcseR_Intercept__ccseR_Intercept
Chain
1
2
3
4
5
6
7
8
9
10

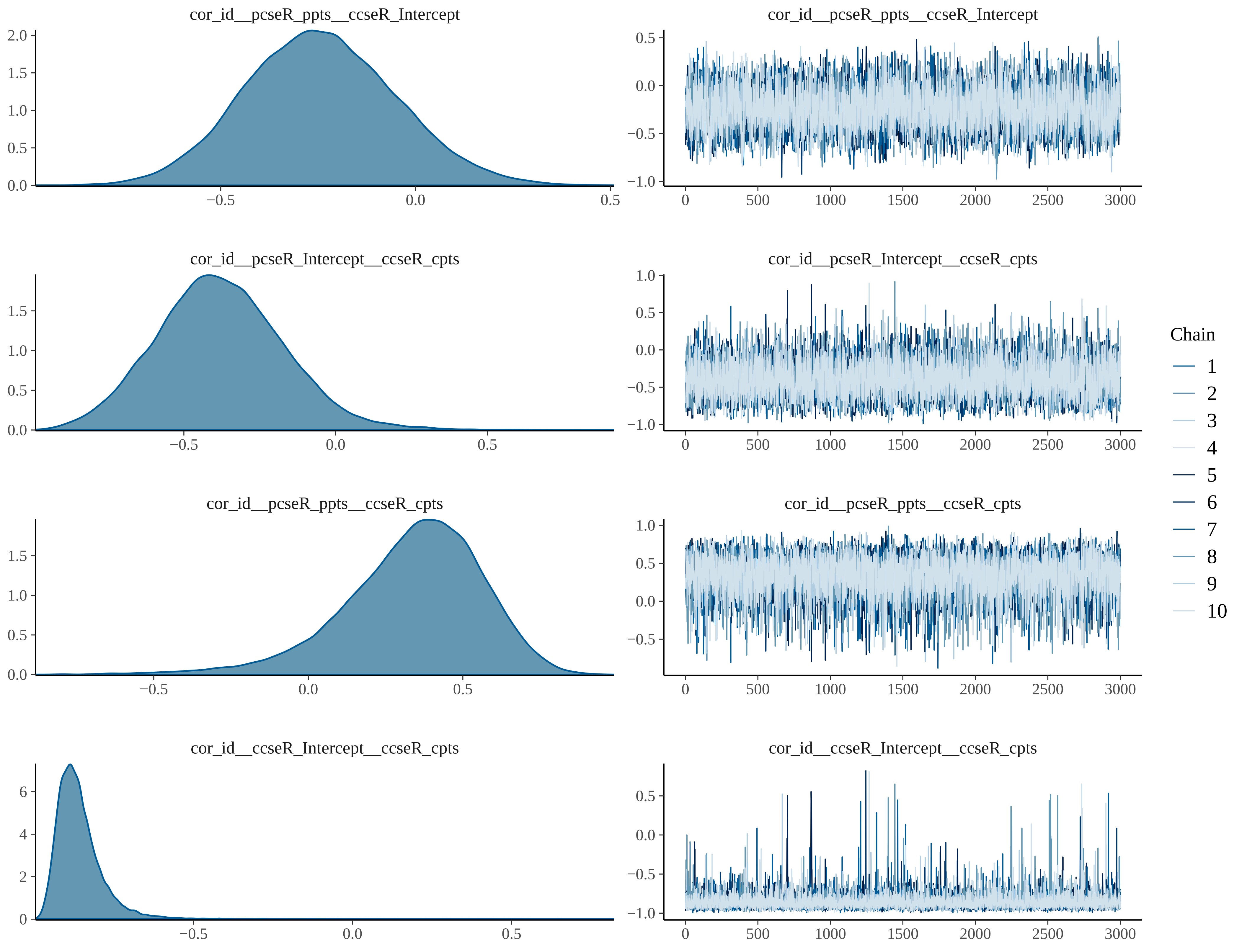
cor_id__pcseR_ppts__ccseR_Intercept
cor_id__pcseR_Intercept__ccseR_cpts
cor_id__pcseR_ppts__ccseR_cpts
cor_id__ccseR_Intercept__ccseR_cpts
Chain
1
2
3
4
5
6
7
8
9
10
